# Enabling endpoint development for interventional clinical trials in individuals with Angelman syndrome: a prospective, longitudinal, observational clinical study (FREESIAS)

**DOI:** 10.1186/s11689-023-09494-w

**Published:** 2023-07-26

**Authors:** Jorrit Tjeertes, Carlos A. Bacino, Terry Jo Bichell, Lynne M. Bird, Mariana Bustamante, Rebecca Crean, Shafali Jeste, Robert W. Komorowski, Michelle L. Krishnan, Meghan T. Miller, David Nobbs, Cesar Ochoa-Lubinoff, Kimberly A. Parkerson, Alexander Rotenberg, Anjali Sadhwani, Mark D. Shen, Lisa Squassante, Wen-Hann Tan, Brenda Vincenzi, Anne C. Wheeler, Joerg F. Hipp, Elizabeth Berry-Kravis

**Affiliations:** 1 F. Hoffmann-La Roche Ltd, Grenzacherstrasse 124, 4070 Basel, Switzerland; 2 Department of Molecular and Human Genetics, Baylor College of Medicine, Houston, TX USA; 3 Texas Children’s Hospital, Houston, TX USA; 4 COMBINEDBrain, Brentwood, TN USA; 5 Department of Pediatrics, University of California San Diego, San Diego, CA USA; 6 Division of Dysmorphology/Genetics, Rady Children’s Hospital, San Diego, CA USA; 7 Ionis Pharmaceuticals Inc, Carlsbad, CA USA; 8 Children’s Hospital Los Angeles, Los Angeles, CA USA; 9 Keck School of Medicine of USC, Los Angeles, CA USA; 10 Biogen, Cambridge, MA USA; 11 Departments of Pediatrics, Division of Developmental-Behavioral Pediatrics, Rush University Medical Center, Chicago, IL USA; 12 Department of Neurology, Boston Children’s Hospital, Harvard Medical School, Boston, MA USA; 13 Department of Psychiatry and Behavioral Services, Boston Children’s Hospital, Harvard Medical School, Boston, MA USA; 14 Carolina Institute for Developmental Disabilities & UNC Neuroscience Center, University of North Carolina, Chapel Hill, NC USA; 15 Division of Genetics and Genomics, Boston Children’s Hospital, Harvard Medical School, Boston, MA USA; 16 Carolina Institute for Developmental Disabilities, Carrboro, NC USA; 17 RTI International, Durham, NC USA; 18 Departments of Pediatrics, Neurological Sciences, Anatomy and Cell Biology, Rush University Medical Center, 1725 W Harrison St, Suite 718, Chicago, IL 60612 USA

**Keywords:** Angelman syndrome, Endpoint development, EEG, Sleep, Digital health technology, Clinical outcome assessments, Natural history, Clinical trials, *UBE3A*

## Abstract

**Background:**

Angelman syndrome (AS) is a rare neurodevelopmental disorder characterized by the absence of a functional *UBE3A* gene, which causes developmental, behavioral, and medical challenges. While currently untreatable, comprehensive data could help identify appropriate endpoints assessing meaningful improvements in clinical trials. Herein are reported the results from the FREESIAS study assessing the feasibility and utility of in-clinic and at-home measures of key AS symptoms.

**Methods:**

Fifty-five individuals with AS (aged < 5 years: *n* = 16, 5–12 years:* n* = 27, ≥ 18 years: *n* = 12; deletion genotype: *n* = 40, nondeletion genotype: *n* = 15) and 20 typically developing children (aged 1–12 years) were enrolled across six USA sites. Several clinical outcome assessments and digital health technologies were tested, together with overnight 19-lead electroencephalography (EEG) and additional polysomnography (PSG) sensors. Participants were assessed at baseline (Clinic Visit 1), 12 months later (Clinic Visit 2), and during intermittent home visits.

**Results:**

The participants achieved high completion rates for the clinical outcome assessments (adherence: 89–100% [Clinic Visit 1]; 76–91% [Clinic Visit 2]) and varied feasibility of and adherence to digital health technologies. The coronavirus disease 2019 (COVID-19) pandemic impacted participants’ uptake of and/or adherence to some measures. It also potentially impacted the at-home PSG/EEG recordings, which were otherwise feasible. Participants achieved Bayley-III results comparable to the available natural history data, showing similar scores between individuals aged ≥ 18 and 5–12 years. Also, participants without a deletion generally scored higher on most clinical outcome assessments than participants with a deletion. Furthermore, the observed AS EEG phenotype of excess delta-band power was consistent with prior reports.

**Conclusions:**

Although feasible clinical outcome assessments and digital health technologies are reported herein, further improved assessments of meaningful AS change are needed. Despite the COVID-19 pandemic, remote assessments facilitated high adherence levels and the results suggested that at-home PSG/EEG might be a feasible alternative to the in-clinic EEG assessments. Taken altogether, the combination of in-clinic/at-home clinical outcome assessments, digital health technologies, and PSG/EEG may improve protocol adherence, reduce patient burden, and optimize study outcomes in AS and other rare disease populations.

**Supplementary Information:**

The online version contains supplementary material available at 10.1186/s11689-023-09494-w.

## Background

Angelman syndrome (AS) is a neurodevelopmental disorder with an estimated incidence of 1 in 22,000 [1–3]. AS is caused by the loss of function of the maternally inherited allele of the ubiquitin-protein ligase E3A (*UBE3A*) gene on Chromosome 15 [4–6]. The most common genetic mechanism underlying AS is a deletion on Chromosome 15q11-q13, which encompasses the *UBE3A* gene, and accounts for approximately 70% of all cases [7]. Other mechanisms include pathogenic variants in *UBE3A*, imprinting defects (ID), and paternal uniparental disomy (UPD) for Chromosome 15 (jointly referred to here as nondeletion AS) [7–9].

AS presents with a broad range of symptoms, including severe-to-profound intellectual disability, lack of speech, ataxia, emotional–behavioral problems, and other medical challenges. These symptoms have a significant impact on individuals with AS and their families, and individuals with AS require 24-h care throughout their lives [10–12]. AS has a significant unmet medical need, whereby there are no approved therapies that directly address the core pathophysiology. Disease management is instead focused on symptomatic treatment and supportive assistance.

To support the development of new therapies, appropriate clinical outcome assessments (COAs) and biomarkers are needed to assess meaningful improvements in individuals with AS. Ideally, such assessments should be noninvasive and minimally burdensome [13]. To inform the development and selection of meaningful endpoints for future interventional trials, recent efforts have been made to classify the disease-defining aspects of AS into disease conceptual models based on input from caregivers and clinical experts [11, 14]. Identified AS-defining domains include seizures, sleep disturbance, maladaptive behaviors, impaired expressive communication, poor fine motor skills, poor gross motor skills, impaired cognition, and limited self-care abilities [11].

Besides COAs, digital health technologies (DHTs) offer promise for remote continuous monitoring [15]. However, the utility of DHTs for individuals with AS has not been comprehensively explored. Cortical activity, assessed by electroencephalography (EEG) is a candidate biomarker for *UBE3A*-related pathophysiology [16–18]. Prolonged, overnight video-EEG recordings assure captured sleep, and thus can provide insight into impaired sleep physiology in AS. Prolonged video-EEG recordings can also enable investigators to quantify epileptiform activity in AS, particularly as seizures and interictal epileptiform discharges are sleep potentiated in many patients with epilepsy [19, 20].

Though several studies have described clinical symptoms in AS [21–30], more comprehensive data are needed for the identification of appropriate COAs to enable endpoint development. In a rare disease population such as AS, innovative, decentralized study designs using a combination of in-clinic, remote, or at-home COAs and DHTs are required to support enrollment, reduce patient burden, and optimize study outcomes.

Here, we describe the 1-year, observational, longitudinal FiRst Endpoint-Enabling Study in Angelman Syndrome (FREESIAS), which was built through a collaborative effort across industry, academia, and together with patient advocacy groups. The primary objective of this study was to evaluate the suitability and feasibility of in-clinic and at-home measures of key AS symptoms for incorporation into AS clinical trials, as well as biomarkers that could capture relevant symptoms and pathophysiology in AS. Remote assessments were also explored to reduce participant and caregiver burden and to increase ecologic validity.

## Methods

### Study design and procedures

**Fig. 1:**
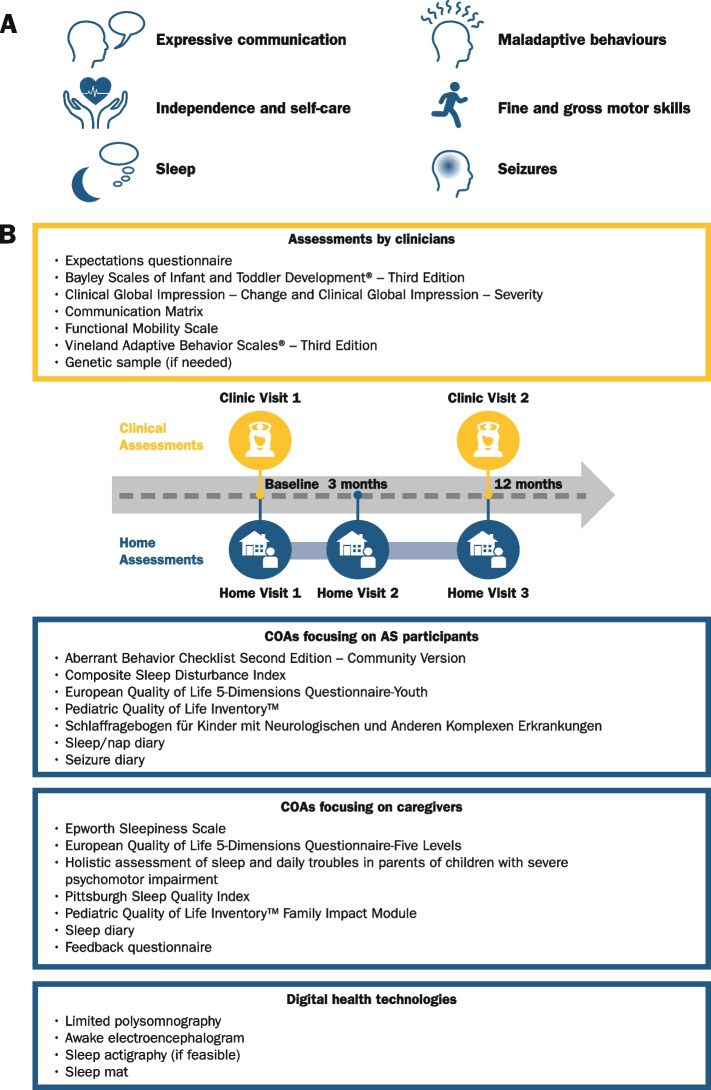
FREESIAS key domains of interest in AS and study design. **A** Key symptoms of interest in AS as identified in a previously published AS disease concept model. **B** FREESIAS study design. The study consisted of two in-clinic visits 12 months apart (Clinic Visit 1 and Clinic Visit 2) and three at-home visits with Home Visit 1 and 3 around the time of Clinic Visit 1 and 2, respectively and Home Visit 2 at 3 months after Home Visit 1. *AS* Angelman syndrome, *COA* clinical outcome assessment

This prospective, observational, longitudinal study (designed following consultations with expert and patient groups) was carried out at six sites in the USA (Boston Children’s Hospital, Boston, Massachusetts; Rady Children’s Hospital, San Diego, California; Rush University Medical Center, Chicago, Illinois; Baylor College of Medicine and Texas Children’s Hospital, Houston, Texas; University of California Los Angeles, Los Angeles, California; University of North Carolina, Chapel Hill, North Carolina) between September 2019 and May 2021 (last study visit). Two clinical visits were planned 12 months apart, as well as a total of three home visits for overnight polysomnography (PSG)/EEG assessments. Data on seizures and sleep were collected continuously via diaries and a sleep mat and in a specific 12-day time window for actigraphy (see Fig. 1). To maintain study adherence, and due to the coronavirus disease 2019 (COVID-19) pandemic, Clinic Visit 2 or Early Withdrawal visits were performed either onsite or remotely. Medical and clinical evaluations such as neurological examinations, weight, height, head circumference measurements, and the assessment of the Bayley Scales of Infant and Toddler Development® – Third Edition (Bayley-III) were conducted in-person. A detailed schedule of activities is in the Additional information (Additional file 1: Schedule of activities). The Bayley-III scale was explored for its potential in investigating the symptoms of AS. To ensure appropriate application, the scale was used according to a standardized administration protocol formulated by a team of psychologists and speech and language pathologists experienced in assessing AS and was applied to all participants regardless of their age [31].

Total estimated duration for completion of all clinical scales at Clinic Visit 1 and Clinic Visit 2 was ~ 8 h but was permitted to be completed over 2 consecutive days. Co-enrollment into other nondrug observational studies was permitted. To reduce burden on study participants and their caregivers, synergy with other ongoing nondrug studies was aimed for. The FREESIAS protocol was prospectively designed to be compatible with the ongoing Angelman Syndrome Natural History Study (AS-NHS; NCT04507997). Overlapping assessments were performed once and data were subsequently shared between both AS-NHS and FREESIAS studies. Participants enrolled in FREESIAS for ≥ 6 months were offered priority screening for future AS clinical drug studies run by F. Hoffmann-La Roche Ltd., Biogen, and Ionis Pharmaceuticals Inc. The FREESIAS study was closed after all participants had been in the study for ≥ 6 months. This was also the minimum duration required for participants to be eligible for a priority voucher for screening in future clinical drug trials.

### Participants

Eligible participants were children with AS aged 1–12 years, adults with AS, and typically developing children (TDC) aged 1–12 years. Children with AS aged 1–12 years were included as they are the likely target population for future interventional clinical trials. Adults with AS were included given the limited natural history data available for this age group. Age-matched TDC, many of whom were siblings of the participants with AS, were to be included to generate reference data for new assessments such as DHTs. The recruitment proceeded relatively easily, with great interest shown by families of individuals with AS. Published evidence indicates that adolescence is a period characterized by great instability and change in AS symptoms as well as clinical heterogeneity [12, 32]. Therefore, adolescents aged 13–17 years were not included in this study.

Key inclusion criteria included a confirmed molecular diagnosis of AS and a caregiver willing to provide written informed consent, comply with study requirements, and accompany participants to clinic visits. Key exclusion criteria included having an unrelated medical condition that might significantly interfere with AS assessment, and current, planned (i.e., within the study duration), or previous participation (i.e., within 4 weeks) in an investigational drug or device trial. Full inclusion and exclusion criteria are in the Additional information (Additional file 1: Inclusion criteria; Additional file 1: Exclusion criteria). Concomitant use of noninvestigational drug products and nonpharmacologic interventions were permitted, with ideally no change throughout the study duration.

For AS participants, all consent was provided by their respective caregiver independent of the AS participants’ age. Assent was provided for TDC aged 1–4 years, with specific assent forms generated for those aged 5–6 years and 7–12 years that also required signatures from their caregiver.

### Clinical outcome assessments focusing on participants

COAs were completed by caregivers or administered by trained evaluators (in clinic or remotely via video conferencing) to the caregiver or participant.

Clinician-reported outcomes (ClinRO) included in the study were the Clinical Global Impression – Severity (CGI-S) scale [33], Vineland Adaptive Behavior Scales® – Third Edition (Vineland-3) [34, 35], Communication Matrix (administered by a trained clinician to caregivers of participants with AS and of TDC) [36], and the Functional Mobility Scale (FMS) [37]. The approach used for CGI-S scale rating is described in the Additional information (Additional file 1: Clinical outcome assessments). Observer-reported outcomes (ObsRO) included Schlaffragebogen für Kinder mit Neurologischen und Anderen Komplexen Erkrankungen (SNAKE; Sleep Questionnaire for Children with Severe Psychomotor Impairment) [38], Composite Sleep Disturbance Index (CSDI) [39, 40], Aberrant Behavior Checklist Second Edition – Community Version (ABC-2-C) [41], Pediatric Quality of Life Inventory™ Generic Core Scales, Version 4.0 (PedsQL™ 4.0 Core) [42], and European Quality of Life 5-Dimensions Questionnaire-Youth Visual Analogue Scale (EQ-5D-Y) [43].

The Bayley-III scale is a standardized developmental assessment for children aged ≤ 42 months although it can be administered to individuals with developmental disabilities beyond the normative age ranges [25, 44]. Due to the substantial impairment of individuals with AS, the Bayley-III was administered outside its normal target age range in this study and used as a performance outcome (PerfO) measure. To reduce cross-site rater-variability, an AS-adapted starting point manual for the Bayley-III was used and is described in the Additional information (Additional file 1: Clinical outcome assessments).

Additional details on the COAs used and a list of the key domains in AS measured by these COAs are in the Additional information (Additional file 1: Clinical outcome assessments). All caregivers were provided an explanation of how to complete the ObsROs by site raters, who themselves were trained by experts.

### Clinical outcome assessments focusing on caregivers

To study the impact of AS on the caregiver and family of participants with AS, the following ObsROs were administered: holistic assessment of sleep and daily troubles in parents of children with severe psychomotor impairment (HOST) [45], Pittsburgh Sleep Quality Index (PSQI) [46], Epworth Sleepiness Scale (ESS) [47], Pediatric Quality of Life Inventory™ Family Impact Module (PedsQL™-FIM) [48], and the European Quality of Life 5-Dimensions Questionnaire-Five Levels (EQ-5D-5L) [49].

### Digital health technologies

This study tested several DHTs to allow for remote and continuous monitoring of study participants (a detailed description of the DHTs used can be found in the Additional information [Additional file 1: Clinical outcome assessments]). DHTs and accompanying manuals were provided to caregivers at the Baseline Visit.

Using a sponsor-provided smartphone, caregivers of participants with AS were asked to register all seizures that occurred during the study in a trial-specific seizure diary and to complete a trial-specific sleep diary every morning. Caregivers were asked to install Emfit® sleep mats under the mattress in the participants’ beds at home, which used the principle of ballistocardiography to register presence in bed as well as several physiologic, behavioral, and sleep-related parameters. Caregivers were asked to place a wearable activity monitor (actigraph; https://actigraphcorp.com/actigraph-link/) on the participant’s nondominant wrist or ankle, abdomen, or chest. The actigraph was worn for up to 10 days at home preceding and up to 2 days after the first home EEG visit.

### Electroencephalography/limited polysomnography

Overnight EEG/limited PSG recordings were performed in the participants’ homes. Data were recorded with Trackit Mk3 (Lifelines Neuro, sampling rate 400 Hz for electrophysiologic signals) and comprised 19 EEG channels (10/20 montage, reference: FC5) [50], and a subset of PSG sensors: one electrocardiogram channel referenced to the EEG reference, two electrooculogram channels (under left eye, and above right eye), two leg electromyography (EMG; left leg, right leg), one abdominal belt, one chin EMG, and one pulse oximeter. Furthermore, the participants were monitored with an infrared camera during sleep to support the data analysis. On the first day of Home Visit 1, 10 min of awake EEG data were analyzed quantitatively following the procedures described by Frohlich et al. (2019) [16] to extract EEG delta-band power.

### Statistical analyses

Due to the exploratory objective of the study, the sample size was based on practical considerations rather than statistical power. The split by age was selected based on the prior studies, which revealed greater rate of developmental gains in children with AS aged 1–4 years compared with those aged 5–12 years across different Bayley-III scales. Continuous endpoints were summarized using descriptive statistics such as means, standard deviations (SD), medians and ranges, and categoric endpoints were summarized using proportions.

## Results

### Recruitment and participant baseline characteristics

A total of 55 participants with AS (aged 1–4 years: *n* = 16, 5–12 years:* n* = 27, ≥ 18 years: *n* = 12) and 20 TDC aged 1–12 years were enrolled over a 12-month period. Seventy-five percent of TDC (*n* = 15) were co-enrolled with a sibling with AS. Fifty-nine of the 75 participants (79%) were enrolled in the first 6 months of the study (Sept 2019–March 2020), prior to the beginning of the COVID-19 pandemic; the last participant was enrolled in September 2020.

All 75 study participants completed Clinic Visit 1 after screening confirmation (there were no screening failures) and 71 participants completed Clinic Visit 2 (51 in clinic; 20 remote). Four participants with AS discontinued the study prematurely and did not complete Clinic Visit 2 (three participants were identified as lost-to-follow-up and one participant had a major protocol deviation [participant enrolled in an investigational drug trial whilst enrolled in FREESIAS]). The mean (± SD) study duration was 362 ± 86 days, ranging from 222–615 days. Nineteen of 55 participants with AS were co-enrolled in the AS-NHS.

Baseline demographics and genetic characteristics are provided in Table 1. The mean (± SD) age at enrollment for participants with AS was 2.9 (± 0.9), 8.3 (± 2.1), and 24.9 (± 5.9) years for the 1–4 year-old, 5–12 year-old, and ≥ 18 year-old groups, respectively, and 6.5 (± 3.4) years for TDC. In total, 40/55 (73%) had deletion AS and 15/55 (27%) had nondeletion AS. Nondeletion genotypes included UPD (*n* = 5; 9%), *UBE3A* mutation (*n* = 5; 9%), and ID (*n* = 4; 7%); one participant classified as either UPD or ID as a more specific diagnosis was not obtained.

**Table 1:** Baseline demographics and genetic characteristics

	**AS deletion (*N* = 40)**	**AS nondeletion (*N* = 15)**	**AS 1–4 years (*N* = 16)**	**AS 5–12 years (*N* = 27)**	**AS ≥ 18 years (*N* = 12)**	**TDC (*N* = 20)**
**Sex**, *n* (%)						
Male	20 (50.0)	11 (73.3)	10 (62.5)	16 (59.3)	5 (41.7)	10 (50.0)
Female	20 (50.0)	4 (26.7)	6 (37.5)	11 (40.7)	7 (58.3)	10 (50.0)
**Mean age,** years ± SD (med; min–max)	10.5 ± 8.7 (7.8; 1–31)	9.9 ± 8.7 (8.7; 3–38)	2.9 ± 0.9 (3.0; 1–5)	8.3 ± 2.1 (8.2; 5–13)	24.9 ± 5.9 (24.7; 18–38)	6.5 ± 3.4 (6.5; 1–13)
**Age group**, *n* (%)						
1–4 years	13 (32.5)	3 (20.0)	16 (100.0)	0 (0.0)	0 (0.0)	9 (45.0)
5–12 years	17 (42.5)	10 (66.7)	0 (0.0)	27 (100.0)	0 (0.0)	11 (55.0)
≥ 18 years	10 (25.0)	2 (13.3)	0 (0.0)	0 (0.0)	12 (100.0)	0 (0.0)
**Race**, *n* (%)						
Asian	1 (2.5)	1 (6.7)	0 (0.0)	1 (3.7)	1 (8.3)	0 (0.0)
Black or African American	3 (7.5)	0 (0.0)	0 (0.0)	1 (3.7)	2 (16.7)	1 (5.0)
White	34 (85.0)	13 (86.7)	14 (87.5)	24 (88.9)	9 (75.0)	16 (80.0)
Multiple	2 (5.0)	1 (6.7)	2 (12.5)	1 (3.7)	0 (0.0)	3 (15.0)
**Ethnicity**, *n* (%)						
Hispanic or Latino	7 (17.5)	5 (33.3)	1 (6.3)	9 (33.3)	2 (16.7)	5 (25.0)
Not Hispanic or Latino	33 (82.5)	10 (66.7)	15 (93.8)	18 (66.7)	10 (83.3)	15 (75.0)
**Mean weight**, kg ± SD (med; min–max)	31.8 ± 21.3 (24.5; 9.8–82.3)	33.1 ± 20.0 (28.2; 14.0–90.8)	13.6 ± 2.6 (14.2; 9.8– 18.1)	28.6 ± 8.4 (27.2; 15.7– 55.0)	64.8 ± 16.0 (62.5; 42.6– 90.8)	23.9 ± 10.5 (21.2; 11.5–47.4)
**Mean height**, cm ± SD (med; min–max)	121.6 ± 26.9* (124.0; 79.0–179.0)	127.1 ± 24.6 (124.0; 91.0–170.0)	92.0 ± 7.6 (92.5; 79.0–106.0)	126.7 ± 12.1 (126.0; 107.0–151.0)	159.6 ± 10.1 (158.0; 144.0–179.0)	118.2 ± 24.9 (117.5; 77.0–165.0)
**Mean BMI**, kg/m^2^ ± SD (med; min–max)	18.5 ± 4.7 (17.2; 13.0–31.1)	19.0 ± 3.9 (18.3; 15.8–31.4)	16.0 ± 1.5 (16.2; 13.0–18.8)	17.5 ± 2.5 (17.2; 13.5–25.1)	24.8 ± 4.8 (24.0; 18.4–31.4)	16.5 ± 1.9 (16.6; 12.4–21.5)
**Mean head circumference**, cm ± SD (med; min–max)	51.1 ± 3.4 (51.5; 45.0–59.0)	51.3 ± 3.6 (51.5; 46.0–58.7)	47.6 ± 1.9 (47.6; 45.0–52.0)	51.6 ± 2.4 (51.5; 47.8–58.0)	54.9 ± 2.4 (54.8; 52.0–59.0)	52.9 ± 2.8^†^ (53.5; 47.0–57.0)
**Genetic variation detected**, *n* (%)						
Class 1 deletion	19 (47.5)	0 (0.0)	9 (56.3)	6 (22.2)	4 (33.3)	N/A
Class 2 deletion	19 (47.5)	0 (0.0)	3 (18.8)	10 (37.0)	6 (50.0)	N/A
Atypical deletion (Class 3 and Class 4)	2 (5.0)	0 (0.0)	1 (6.3)	1 (3.7)	0 (0.0)	N/A
ID	0 (0.0)	4 (26.7)	0 (0.0)	3 (11.1)	1 (8.3)	N/A
*UBE3A* mutation	0 (0.0)	5 (33.3)	1 (6.3)	4 (14.8)	0 (0.0)	N/A
UPD	0 (0.0)	5 (33.3)	2 (12.5)	3 (11.1)	0 (0.0)	N/A
Not defined (UPD or ID)	0 (0.0)	1 (6.7)	0 (0.0)	0 (0.0)	1 (8.3)	N/A

*AS* Angelman syndrome, *BMI* Body mass index, *ID* Imprinting defect, *Max* Maximum, *Med* Median, *Min* Minimum, *N/A* Not assessed, *SD* Standard deviation, *TDC* Typically developing children, *UBE3A* Ubiquitin-protein ligase E3A, *UPD* Uniparental disomy

^*^n = 39, ^†^*n* = 19

At baseline, gastrointestinal disorders were reported by 87% of those with AS (48/55), the most common condition being gastroesophageal reflux disease in 60% (33/55) of participants with AS followed by constipation (55%; 30/55). Eye disorders were reported in 47% (26/55) of participants with AS, with strabismus being the most commonly reported (40%; 22/55). Psychiatric disorders were reported in 66% (36/55) of participants with AS including insomnia (15/55) and anxiety (7/55; Table S1). As part of the seizure history assessment a total of 75% (41/55) of participants with AS had epilepsy including generalized epilepsy (34%; 14/41); focal epilepsy (24%; 10/41); and combined generalized and focal epilepsy (39%; 16/41); the epilepsy type of one participant was unknown (Table S2).

Even though no formal anchor was provided (see Discussion), ratings were provided by placing the participant in the context of AS i.e., “mildly ill” when compared to the general AS population known to the expert clinician. The most common CGI-S response for participants with AS aged 1–4 years (31%; 5/16) was a score of 3 (*Mildly ill*), while the most common response for participants with AS aged 5–12 years (37%; 10/27) was a score of 4 (*Moderately ill*). Among participants with AS aged ≥ 18 years, an equal number responded with a score of 4 (*Moderately ill*, 42%; 5/12) and 5 (*Markedly ill*, 42%; 5/12). Participants with deletion AS were most frequently rated with a score of 4 (35%; 14/40), whereas those with nondeletion AS most frequently received a score of 3 (47%; 7/15). Overall, participants with deletion AS were more frequently rated with a score of 5 (*Markedly ill*, 28%; 11/40) or 6 (*Severely ill*, 10%; 4/40), compared with participants with nondeletion AS (*Markedly ill*, 13%; 2/15 or *Severely ill*, 0%; 0/15).

### Feasibility and adherence of COAs, DHTs, and overnight EEG

#### Clinical outcome assessments

An overview of the completion rate from all COAs can be found in Table 2. A completion rate of 89–100% was obtained for COAs at Clinic Visit 1 in participants with AS. At Clinic Visit 2, Early Withdrawal, or Remote Visits, the completion rate was 76–91% apart from Bayley-III, which had a lower completion rate of 62%, since it requires in-person administration (which was not possible at some sites in some cases due to COVID-19 pandemic restrictions). TDC demonstrated adherence of 95–100% during both Clinic Visit 1 and 2.

**Table 2:** Overall completion rates for clinical outcome assessments

	**AS (*N* = 55)**		**TDC (*N* = 20)**	
	**Clinic Visit 1**, *n* (%)	Clinic Visit 2,* *n* (%)	**Clinic Visit 1**, *n* (%)	Clinic Visit 2,* *n* (%)
**Bayley-III**	55 (100.0)	34 (61.9)	N/A	N/A
**Vineland-3**	55 (100.0)	50 (90.9)	20 (100.0)	20 (100.0)
**Communication Matrix**	52 (94.5)	50 (90.9)	19 (95.0)	20 (100.0)
**SNAKE**	49 (89.1)	43 (78.2)	N/A	N/A
**CSDI**	49 (89.1)	42 (76.4)	N/A	N/A
**ABC-2-C**	50 (90.9)	44 (80.0)	N/A	N/A
**CGI-S**	55 (100.0)	49 (89.1)	N/A	N/A
**FMS**	55 (100.0)	48 (87.3)	20 (100.0)	20 (100.0)
**PedsQL™ 4.0 Core**	53 (96.4)	44 (80.0)	N/A	N/A
**EQ-5D-Y**	49 (89.1)	43 (78.2)	N/A	N/A
**HOST**	50 (90.9)	43 (78.2)	N/A	N/A
**PedsQL™-FIM**	53 (96.4)	44 (80.0)	N/A	N/A
**ESS**	50 (90.9)	42 (76.4)	N/A	N/A
**PSQI**	50 (90.9)	42 (76.4)	N/A	N/A
**EQ-5D-5L**	49 (89.1)	42 (76.4)	N/A	N/A

Percentages are derived based on the number of participants who completed assessments at that visit

*ABC-2-C* Aberrant Behavior Checklist Second Edition – Community Version, *AS* Angelman syndrome, *Bayley-III* Bayley Scales of Infant and Toddler Development® – Third Edition, *CGI-S* Clinical Global Impression – Severity, *COVID-19* coronavirus disease 2019, *CSDI* Composite Sleep Disturbance Index, *EQ-5D-5L* European Quality of Life 5-Dimensions Questionnaire-Five Levels, *EQ-5D-Y* European Quality of Life 5-Dimensions Questionnaire-Youth, *ESS* Epworth Sleepiness Scale, *FMS* Functional Mobility Scale, *HOST* holistic assessment of sleep and daily troubles in parents of children with severe psychomotor impairment, *N/A* not assessed, *PedsQL™ 4.0 Core* Pediatric Quality of Life Inventory™ Generic Core Scales, Version 4.0, *PedsQL™-FIM* Pediatric Quality of Life Inventory™ Family Impact Module, *PSQI* Pittsburgh Sleep Quality Index, *SNAKE* Schlaffragebogen für Kinder mit Neurologischen und Anderen Komplexen Erkrankungen (Sleep Questionnaire for Children with Severe Psychomotor Impairment), *TDC* typically developing children, *Vineland-3* Vineland Adaptive Behavior Scales® – Third Edition

^*^Clinic Visit 2 includes data captured during Clinic Visit 2, Early Withdrawal, Remote Visit, and out-of-time window (due to COVID-19)

Baseline data for all assessments (presented as raw scores, unless stated otherwise) by genotype and age are shown in Table 3. While many assessments showed different levels of mean variability, participants with nondeletion AS tended to have higher scores (i.e., higher performance/less impairment) than participants with deletion AS across assessments. Age-dependent differences were also evident, with those aged 5–12 years generally having higher scores compared with those aged 1–4 years. There were largely no further gains in scores for those aged ≥ 18 years. Cross-sectional comparison of participants with AS aged ≥ 18 years showed similar characteristics to those aged 5–12 years.

**Table 3:** Baseline COA data by genotype and age

Scales	AS 1–4 years	AS 5–12 years	AS ≥ 18 years	AS deletion	AS nondeletion	TDC 1–4 years	TDC 5–12 years
**Bayley-III raw score,** *n*, mean ± SD, range							
Cognitive	*n* = 16	*n* = 26	*n* = 12	*n* = 40	*n* = 14	N/A	N/A
	35.5 ± 7.4	49.9 ± 11.1	50.9 ± 11.6	41.5 ± 9.6	58.3 ± 10.0	N/A	N/A
	23.0–52.0	29.0–79.0	30.0–66.0	23.0–63.0	41.0–79.0	N/A	N/A
Expressive Communication	*n* = 15	*n* = 27	*n* = 12	*n* = 39	*n* = 15	N/A	N/A
	9.0 ± 3.7	11.9 ± 4.3	15.8 ± 5.1	10.9 ± 4.3	14.7 ± 5.5	N/A	N/A
	2.0–17.0	6.0–20.0	7.0–27.0	2.0–20.0	7.0–27.0	N/A	N/A
Receptive Communication	*n* = 16	*n* = 25	*n* = 12	*n* = 39	*n* = 14	N/A	N/A
	12.9 ± 4.2	17.4 ± 5.6	19.3 ± 7.0	14.3 ± 4.4	22.6 ± 5.8	N/A	N/A
	7.0–23.0	9.0–28.0	11.0–33.0	7.0–28.0	12.0–33.0	N/A	N/A
Fine Motor	*n* = 15	*n* = 26	*n* = 12	*n* = 39	*n* = 14	N/A	N/A
	24.1 ± 3.8	29.6 ± 7.7	35.3 ± 8.0	27.0 ± 6.5	35.9 ± 7.7	N/A	N/A
	18.0–31.0	16.0–43.0	25.0–49.0	16.0–44.0	21.0–49.0	N/A	N/A
Gross Motor	*n* = 16	*n* = 27	*n* = 10	*n* = 38	*n* = 15	N/A	N/A
	36.6 ± 10.7	49.3 ± 6.7	48.4 ± 7.6	42.8 ± 10.1	51.5 ± 6.5	N/A	N/A
	15.0–54.0	29.0–66.0	29.0–55.0	15.0–54.0	43.0–66.0	N/A	N/A
**Vineland-3 raw score,** *n*, mean ± SD, range							
Expressive Communication	*n* = 16	*n* = 27	*n* = 11	*n* = 40	*n* = 14	*n* = 9	*n* = 11
	11.3 ± 7.2	16.3 ± 8.4	16.0 ± 8.9	12.6 ± 7.1	20.9 ± 8.8	70.6 ± 31.0	96.7 ± 1.4
	4.0–28.0	5.0–40.0	10.0–38.0	4.0–40.0	10.0–38.0	17.0–94.0	95.0–98.0
Receptive Communication	*n* = 16	*n* = 27	*n* = 12	*n* = 40	*n* = 15	*n* = 9	*n* = 11
	16.7 ± 11.9	29.2 ± 13.5	29.6 ± 15.9	21.0 ± 13.4	38.1 ± 9.5	56.8 ± 17.2	75.6 ± 2.1
	1.0–42.0	9.0–62.0	11.0–55.0	1.0–62.0	25.0–55.0	27.0–72.0	72.0–78.0
Fine Motor	*n* = 16	*n* = 27	*n* = 12	*n* = 40	*n* = 15	*n* = 9	*n* = 8
	14.0 ± 6.0	24.7 ± 7.0	25.1 ± 8.1	19.0 ± 7.2	28.9 ± 7.3	39.8 ± 11.6	67.0 ± 1.6
	5.0–27.0	14.0–44.0	16.0–38.0	5.0–36.0	18.0–44.0	18.0–51.0	64.0–68.0
Gross Motor	*n* = 16	*n* = 27	*n* = 11	*n* = 39	*n* = 15	*n* = 9	*n* = 8
	21.6 ± 15.1	48.2 ± 13.3	39.2 ± 15.8	34.4 ± 17.4	49.1 ± 16.3	70.7 ± 17.6	85.1 ± 1.4
	2.0–56.0	17.0–77.0	11.0–58.0	2.0–58.0	25.0–77.0	38.0–83.0	83.0–86.0
**Communication Matrix total score,** *n*, mean ± SD, range	*n* = 13	*n* = 26	*n* = 11	*n* = 38	*n* = 12	*n* = 8	*n* = 11
	38.5 ± 19.9	67.7 ± 26.0	56.6 ± 19.0	48.5 ± 18.3	86.6 ± 25.3	120.6 ± 38.5	145.8 ± 12.8
	5.0–72.0	33.0–135.0	32.0–100.0	5.0–109.0	46.0–135.0	55.0–160.0	126.0–160.0
**SNAKE,** *n*, mean ± SD, range							
Disturbances going to sleep	*n* = 13	*n* = 26	*n* = 9	*n* = 36	*n* = 12	N/A	N/A
	9.0 ± 1.6	11.8 ± 3.5	9.2 ± 2.7	10.2 ± 3.1	11.7 ± 3.2	N/A	N/A
	7.0–12.0	7.0–19.0	6.0–14.0	6.0–18.0	8.0–19.0	N/A	N/A
Disturbances remaining asleep	*n* = 12	*n* = 25	*n* = 9	*n* = 36	*n* = 10	N/A	N/A
	15.1 ± 3.3	13.3 ± 3.4	12.8 ± 3.0	13.6 ± 3.1	14.0 ± 4.4	N/A	N/A
	10.0–20.0	5.0–19.0	10.0–18.0	8.0–19.0	5.0–20.0	N/A	N/A
Arousal disorders	*n* = 13	*n* = 25	*n* = 9	*n* = 35	*n* = 12	N/A	N/A
	9.5 ± 2.5	9.0 ± 2.7	9.1 ± 3.7	9.3 ± 3.0	8.7 ± 2.1	N/A	N/A
	6.0–14.0	6.0–15.0	6.0–17.0	6.0–17.0	6.0–12.0	N/A	N/A
Daytime sleepiness	*n* = 12	*n* = 23	*n* = 10	*n* = 32	*n* = 13	N/A	N/A
	8.6 ± 1.6	5.9 ± 2.1	6.2 ± 3.1	6.6 ± 2.4	6.9 ± 2.7	N/A	N/A
	6.0–11.0	3.0–10.0	3.0–11.0	3.0–11.0	3.0–11.0	N/A	N/A
Daytime behavioral disorders	*n* = 13	*n* = 26	*n* = 9	*n* = 36	*n* = 12	N/A	N/A
	9.2 ± 2.3	9.9 ± 3.3	8.3 ± 2.6	9.3 ± 2.8	9.8 ± 3.4	N/A	N/A
	6.0–13.0	4.0–15.0	5.0–12.0	5.0–15.0	4.0–15.0	N/A	N/A
**CSDI,** *n*, mean ± SD, range	*n* = 12	*n* = 25	*n* = 10	*n* = 35	*n* = 12	N/A	N/A
	5.3 ± 2.4	5.6 ± 2.6	6.0 ± 2.4	5.3 ± 2.5	6.4 ± 2.3	N/A	N/A
	0.0–8.0	2.0–11.0	2.0–9.0	0.0–11.0	2.0–10.0	N/A	N/A
**ABC-2-C,** *n*, mean ± SD, range							
Irritability	*n* = 13	*n* = 26	*n* = 11	*n* = 37	*n* = 13	N/A	N/A
	4.2 ± 5.5	9.6 ± 8.7	10.0 ± 7.1	7.8 ± 6.8	9.9 ± 10.6	N/A	N/A
	0.0–19.0	1.0–39.0	0.0–23.0	0.0–27.0	0.0–39.0	N/A	N/A
Social withdrawal	*n* = 13	*n* = 26	*n* = 11	*n* = 37	*n* = 13	N/A	N/A
	5.9 ± 5.8	6.4 ± 4.3	4.7 ± 4.8	6.7 ± 4.8	3.7 ± 4.1	N/A	N/A
	0.0–20.0	1.0–16.0	0.0–17.0	0.0–20.0	0.0–16.0	N/A	N/A
Stereotypic behavior	*n* = 13	*n* = 26	*n* = 11	*n* = 37	*n* = 13	N/A	N/A
	4.9 ± 4.7	6.5 ± 5.2	1.9 ± 2.2	5.6 ± 4.7	3.5 ± 5.0	N/A	N/A
	0.0–14.0	0.0–18.0	0.0–6.0	0.0–16.0	0.0–18.0	N/A	N/A
Hyperactivity/ noncompliance	*n* = 13	*n* = 26	*n* = 11	*n* = 37	*n* = 13	N/A	N/A
	9.9 ± 11.6	22.4 ± 11.3	15.7 ± 8.3	18.1 ± 12.2	16.5 ± 11.2	N/A	N/A
	0.0–39.0	5.0–40.0	1.0–25.0	0.0–39.0	1.0–40.0	N/A	N/A
Inappropriate speech	*n* = 13	*n* = 26	*n* = 11	*n* = 37	*n* = 13	N/A	N/A
	0.4 ± 1.4	1.1 ± 2.7	1.6 ± 2.4	0.8 ± 1.8	1.5 ± 3.6	N/A	N/A
	0.0–5.0	0.0–12.0	0.0–6.0	0.0–7.0	0.0–12.0	N/A	N/A
**FMS**, *n* (%)							
** 5 m**	*n* = 16	*n* = 27	*n* = 12	*n* = 40	*n* = 15	*n* = 9	*n* = 11
C – Crawling	3 (18.8)	0 (0.0)	1 (8.3)	4 (10.0)	0 (0.0)	0 (0.0)	0 (0.0)
N – Does not apply	2 (12.5)	0 (0.0)	0 (0.0)	2 (5.0)	0 (0.0)	0 (0.0)	0 (0.0)
1 – Wheelchair	2 (12.5)	1 (3.7)	1 (8.3)	4 (10.0)	0 (0.0)	0 (0.0)	0 (0.0)
2 –Walker/frame	3 (18.8)	1 (3.7)	1 (8.3)	4 (10.0)	1 (6.7)	0 (0.0)	0 (0.0)
3 – Crutches	0 (0.0)	0 (0.0)	0 (0.0)	0 (0.0)	0 (0.0)	0 (0.0)	0 (0.0)
4 – Sticks	1 (6.3)	0 (0.0)	0 (0.0)	1 (2.5)	0 (0.0)	0 (0.0)	0 (0.0)
5 – Independent: level surfaces	1 (6.3)	13 (48.1)	3 (25.0)	11 (27.5)	6 (40.0)	0 (0.0)	0 (0.0)
6 – Independent: all surfaces	4 (25.0)	12 (44.4)	6 (50.0)	14 (35.0)	8 (53.3)	9 (100.0)	11 (100.0)
** 50 m**	*n* = 16	*n* = 27	*n* = 12	*n* = 40	*n* = 15	*n* = 9	*n* = 11
N – Does not apply	4 (25.0)	0 (0.0)	0 (0.0)	4 (10.0)	0 (0.0)	0 (0.0)	0 (0.0)
1 – Wheelchair	4 (25.0)	2 (7.4)	2 (16.7)	8 (20.0)	0 (0.0)	0 (0.0)	0 (0.0)
2 – Walker/frame	2 (12.5)	1 (3.7)	1 (8.3)	2 (5.0)	2 (13.3)	0 (0.0)	0 (0.0)
3 – Crutches	0 (0.0)	0 (0.0)	0 (0.0)	0 (0.0)	0 (0.0)	0 (0.0)	0 (0.0)
4 – Sticks	1 (6.3)	0 (0.0)	0 (0.0)	1 (2.5)	0 (0.0)	0 (0.0)	0 (0.0)
5 – Independent: level surfaces	3 (18.8)	16 (59.3)	4 (33.3)	16 (40.0)	7 (46.7)	0 (0.0)	0 (0.0)
6 – Independent: all surfaces	2 (12.5)	8 (29.6)	5 (41.7)	9 (22.5)	6 (40.0)	9 (100.0)	11 (100.0)
** 500 m**	*n* = 16	*n* = 27	*n* = 12	*n* = 40	*n* = 15	*n* = 9	*n* = 11
N – Does not apply	5 (31.3)	0 (0.0)	0 (0.0)	4 (10.0)	1 (6.7)	2 (22.2)	0 (0.0)
1 – Wheelchair	5 (31.3)	5 (18.5)	3 (25.0)	10 (25.0)	3 (20.0)	0 (0.0)	0 (0.0)
2 – Walker/frame	3 (18.8)	0 (0.0)	1 (8.3)	3 (7.5)	1 (6.7)	0 (0.0)	0 (0.0)
3 – Crutches	0 (0.0)	0 (0.0)	0 (0.0)	0 (0.0)	0 (0.0)	0 (0.0)	0 (0.0)
4 – Sticks	0 (0.0)	2 (7.4)	0 (0.0)	0 (0.0)	2 (13.3)	0 (0.0)	0 (0.0)
5 – Independent: level surfaces	2 (12.5)	13 (48.1)	4 (33.3)	17 (42.5)	2 (13.3)	0 (0.0)	0 (0.0)
6 – Independent: all surfaces	1 (6.3)	7 (25.9)	4 (33.3)	6 (15.0)	6 (40.0)	7 (77.8)	11 (100.0)
**PedsQL™ 4.0 Core,** *n* mean ± SD, range	*n* = 13	*n* = 26	*n* = 11	*n* = 37	*n* = 13	N/A	N/A
	55.9 ± 12.0	53.6 ± 17.1	51.8 ± 17.2	53.1 ± 15.7	55.8 ± 16.5	N/A	N/A
	36.9–72.9	21.4–84.5	25.0–81.0	25.0–84.5	21.4–79.8	N/A	N/A
**EQ-5D-Y VAS**, *n* mean ± SD, range	*n* = 13	*n* = 24	*n* = 11	*n* = 35	*n* = 13	N/A	N/A
	83.1 ± 9.5	81.9 ± 12.1	89.2 ± 14.7	84.4 ± 12.0	82.5 ± 13.0	N/A	N/A
	70.0–100.0	50.0–98.0	62.0–100.0	55.0–100.0	50.0–100.0	N/A	N/A
**CGI-S,** *n* (%)							
	*n* = 16	*n* = 27	*n* = 12	*n* = 40	*n* = 15	N/A	N/A
1 − *Normal, not at all ill*	1 (6.3)	2 (7.4)	0 (0.0)	3 (7.5)	0 (0.0)	N/A	N/A
2 − *Borderline ill*	2 (12.5)	2 (7.4)	1 (8.3)	4 (10.0)	1 (6.7)	N/A	N/A
3 − *Mildly ill*	5 (31.3)	6 (22.2)	0 (0.0)	4 (10.0)	7 (46.7)	N/A	N/A
4 − *Moderately ill*	4 (25.0)	10 (37.0)	5 (41.7)	14 (35.0)	5 (33.3)	N/A	N/A
5 − *Markedly ill*	3 (18.8)	5 (18.5)	5 (41.7)	11 (27.5)	2 (13.3)	N/A	N/A
6 − *Severely ill*	1 (6.3)	2 (7.4)	1 (8.3)	4 (10.0)	0 (0.0)	N/A	N/A
**HOST,** *n*, mean ± SD, range							
Sleep disturbances	*n* = 13	*n* = 26	*n* = 11	*n* = 37	*n* = 13	N/A	N/A
	16.0 ± 5.5	15.0 ± 5.0	13.7 ± 7.1	14.7 ± 5.5	15.9 ± 5.8	N/A	N/A
	8.0–24.0	5.0–25.0	5.0–25.0	5.0–25.0	5.0–25.0	N/A	N/A
Impairment of physical/mental functioning	*n* = 13	*n* = 26	*n* = 11	*n* = 37	*n* = 13	N/A	N/A
	12.5 ± 4.3	12.1 ± 5.6	10.7 ± 4.0	11.8 ± 4.7	12.3 ± 5.6	N/A	N/A
	6.0–22.0	5.0–25.0	5.0–16.0	5.0–23.0	5.0–25.0	N/A	N/A
Impairment of social functioning	*n* = 13	*n* = 26	*n* = 11	*n* = 37	*n* = 13	N/A	N/A
	9.5 ± 3.3	11.1 ± 3.5	11.4 ± 3.5	10.7 ± 3.3	11.0 ± 4.0	N/A	N/A
	4.0–15.0	4.0–20.0	7.0–18.0	4.0–18.0	5.0–20.0	N/A	N/A
Impairment of working ability	*n* = 13	*n* = 26	*n* = 11	*n* = 37	*n* = 13	N/A	N/A
	3.5 ± 2.2	4.0 ± 1.8	3.5 ± 1.4	3.8 ± 1.9	3.6 ± 1.6	N/A	N/A
	2.0–8.0	2.0–10.0	2.0–7.0	2.0–10.0	2.0–7.0	N/A	N/A
**PSQI,** *n*, mean ± SD, range	*n* = 12	*n* = 24	*n* = 10	*n* = 35	*n* = 11	N/A	N/A
	9.3 ± 4.8	7.2 ± 4.1	7.5 ± 3.4	7.7 ± 4.3	8.3 ± 3.9	N/A	N/A
	5.0–17.0	1.0–17.0	3.0–12.0	1.0–17.0	5.0–17.0	N/A	N/A
**ESS**, *n*, mean ± SD, range	*n* = 13	*n* = 26	*n* = 11	*n* = 37	*n* = 13	N/A	N/A
	8.2 ± 4.2	8.1 ± 4.2	7.7 ± 4.0	8.0 ± 4.4	8.3 ± 3.0	N/A	N/A
	3.0–16.0	1.0–16.0	2.0–14.0	1.0–16.0	3.0–11.0	N/A	N/A
**EQ-5D-5L VAS,** *n*, mean ± SD, range	*n* = 13	*n* = 25	*n* = 11	*n* = 36	*n* = 13	N/A	N/A
	82.7 ± 9.3	82.2 ± 9.5	87.6 ± 8.6	84.3 ± 9.6	81.4 ± 8.4	N/A	N/A
	60.0–90.0	60.0–95.0	75.0–100.0	60.0–100.0	70.0–95.0	N/A	N/A
**PedsQL™-FIM,** *n*, mean ± SD, range	*n* = 13	*n* = 26	*n* = 11	*n* = 37	*n* = 13	N/A	N/A
	56.9 ± 17.8	54.1 ± 20.1	58.6 ± 13.0	56.2 ± 17.1	54.7 ± 20.8	N/A	N/A
	26.4–83.3	10.4–98.6	46.5–94.4	26.4–98.6	10.4–76.4	N/A	N/A

*ABC-2-C* Aberrant Behavior Checklist Second Edition – Community Version, *AS* Angelman syndrome, *Bayley-III* Bayley Scales of Infant and Toddler Development® – Third Edition, *CGI-S* Clinical Global Impression – Severity, *COA* clinical outcome assessment, *CSDI* Composite Sleep Disturbance Index, *EQ-5D-5L VAS* European Quality of Life 5-Dimensions Questionnaire-Five Levels Visual Analogue Scale, *EQ-5D-Y VAS* European Quality of Life 5-Dimensions Questionnaire-Youth Visual Analogue Scale, *ESS* Epworth Sleepiness Scale, *FMS* Functional Mobility Scale, *HOST* holistic assessment of sleep and daily troubles in parents of children with severe psychomotor impairment, *N/A* not accessed, *PedsQL™ 4.0 Core* Pediatric Quality of Life Inventory™ Generic Core Scales, Version 4.0, *PedsQL™-FIM* Pediatric Quality of Life Inventory™ Family Impact Module, *PSQI* Pittsburgh Sleep Quality Index, *SD* Standard deviation, *SNAKE* Schlaffragebogen für Kinder mit Neurologischen und Anderen Komplexen Erkrankungen (Sleep Questionnaire for Children with Severe Psychomotor Impairment), *TDC* Typically developing children, *Vineland-3* Vineland Adaptive Behavior Scales® – Third Edition

### Digital health technologies

#### Seizure diary

A total of 631 unique seizure events among 18 participants were reported via the seizure diary throughout the study. Some caregivers provided incomplete seizure information. For example, some answers were left blank for recovery time (17%; 105/631 events), type of seizure (14%; 88/631 events), and seizure duration (12%; 74/631 events). A summary of the characteristics of participants and reported seizures is provided in Table S3.

Seizure diary adherence could not be determined as no specific means to track confirmations about the absence of seizures within the app was available. Consequently, it is likely that seizures were underreported. For most participants reporting seizures, there were more seizures reported in the first 90 days of participation than in the last 90 days (see Fig. S1). This suggests that adherence to the seizure diaries declined over time, since there is no obvious reason to assume fewer seizures in the second part of the observation period.

The frequency of seizures reported through the seizure diary was consistent with that reported at baseline in most participants who completed the diary (61%; 11/18). There were 24 individuals out of 41 (59%) with a history of epilepsy reported at baseline who did not report seizures through the seizure diary app nor as an adverse event during the observation period. Three individuals that did not report a history of epilepsy at baseline developed a first seizure during the study (one participant with nondeletion AS age 4.4 years; two participants with deletion AS ages 1.6 and 2.2 years).

### Sleep diary

**Fig. 2:**
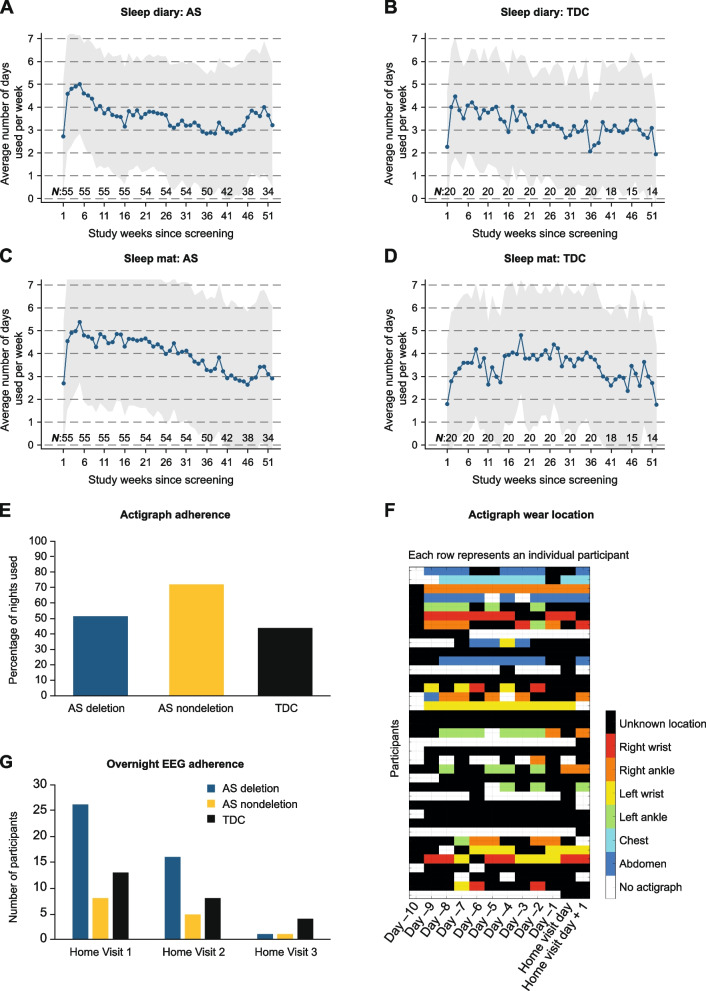
Adherence/completed assessments for sleep diaries, sleep mat, actigraph, and overnight EEG. Adherence to sleep diary completion in participants with AS (**A**) and TDC (**B**). Adherence was defined as the percentage of days within the 52-week observational period in which a diary entry was recorded. Adherence to the sleep mat in participants with AS (**C**) and TDC (**D**). Shaded area in graphs A–D represents ± 1 SD. Sleep mat usage was defined as the percentage of days per week within the 52-week observational period in which a sleep mat recording was started. The number of participants included in the adherence calculation for both the sleep diary and sleep mat decreased over the 52 weeks due to either withdrawal, rollover to clinical drug trials, or early completion (see “*N*” above x-axis). **E** The percentage of nights that the actigraph was used in the Actigraphy Sleep Monitoring Period for those participants for which any data were collected (AS deletion *n* = 31; AS nondeletion *n* = 11; TDC *n* = 13). **F** Representation of wearing location of the actigraph during the home monitoring period with unknown location (black); right wrist (red); right ankle (orange); left wrist (yellow); left ankle (green); chest (light blue); abdomen (dark blue); and no actigraphy (black). The actigraph was worn for up to 10 days at home preceding and up to 2 days after the first home EEG visit. **G** Number of participants who completed overnight EEG recordings with usable data of at least 5 h duration at Home Visit 1, 2, and 3. *AS* Angelman syndrome, *EEG* Electroencephalogram, *SD* Standard deviation*, TDC* Typically developing children

Caregivers were asked to report every morning on the participants’ sleep during the study. Caregiver adherence to the sleep diary was defined as the percentage of days per week within the 52-week observational period in which they completed the diary. The number of participants included in the adherence calculation decreased over the 52 weeks due either to withdrawal or early completion. Mean adherence was 52% (interquartile range [IQR]: 34–72%) for participants with AS and 47% (IQR: 34–70%) for TDC (see Fig. 2A, B). Mean adherence was higher in the first 26 weeks than in weeks 27–52, respectively (participants with AS: 56% [IQR: 42–75%] vs. 48% [IQR: 20–74%]; TDC: 51% [IQR: 43–73%] vs. 42% [IQR: 16–67%]).

### Sleep mat

A sleep mat placed under each participant’s mattress was used to record sleep daily over the course of the study. The actual use period of the sleep mat was defined as the percentage of days per week in which a sleep mat recording was started and was calculated for every week of the 52-week observational period. The number of participants included in the calculation decreased over the 52 weeks due to either withdrawal or early completion (see Fig. 2C, D). The mean (IQR) actual use period was 59% (42–87) for participants with AS and 53% (32–74) for TDC. For participants with AS, mean (IQR) adherence was higher in the first 26 weeks than in weeks 27–52 (63% [43–90] vs. 56% [30–86]); this was not the case for TDC (53% [33–81] vs. 55% [37–76], respectively). Reasons for nonuse were not collected systematically, but the following reasons were identified by the investigational sites: participant interfered with device (*n* = 5); participant slept in different location (*n* = 3); caregiver overwhelmed and did not have time (*n* = 4); sleep mat made bed uncomfortable (*n* = 3); sleep mat caused participant distress (*n* = 2); and sleep mat was incompatible with bed (*n* = 1).

### Sleep actigraphy

Sleep actigraphy recordings were planned for each night between 10 days before, up to, and including Home Visit 1, and up to 2 days following Home Visit 1. Due to COVID-19, 20/75 participants did not have a first home visit and therefore were not asked to use the actigraph. Participant adherence to sleep actigraphy was calculated for the remaining 55 participants, defined as the percentage of days within this period on which the actigraph was worn for ≥ 30 min. Mean (IQR) adherence during this period was 56% (0–100) for participants with AS and 44% (2–98) for TDC (see Fig. 2E). Participants were given the option of wearing the actigraph on either the wrist or ankle, or in a pocket on the chest or abdomen and caregivers were asked to enter the wear position in the sleep diary. Some caregivers recorded different positions for different nights (see Fig. 2F). For 16/55 (29%) participants with AS, no actigraph recording was made during Home Visit 1. Reasons for nonadherence were not collected systematically, but the following were noted based on site follow-up with caregivers: not feasible for the caregiver (*n* = 3); participant did not tolerate actigraph (*n* = 3); and participant interfered with device (*n* = 1).

### Overnight electroencephalography at home

Ninety out of 225 (40%) of the planned home visits were attempted and led to 85 successful overnight EEG recordings, defined as > 5 h of data per EEG recording; mean (± SD) duration of 14 (± 4.6) hours. The EEG recording period was purposefully ~ 14 h long on an average, as it would start in the afternoon to capture participants’ awake EEG data. Home visits were not carried out for reasons related to the COVID-19 pandemic, e.g., it was legally not possible, the EEG vendor was not able to provide service, or the caregiver did not agree to the home visit due to COVID-19. EEG recordings were obtained from 47/75 participants at Home Visit 1, including 13 TDC and 34 individuals with AS (Fig. 2G; Table S4).

The quality of the scalp EEG data was considered sufficient by EEG experts (neuroscientists and neurologists) in most cases to perform quantitative analyses and to identify waking and sleep background elements, epileptiform discharges (spikes and sharp waves), and epileptic seizures.

### FREESIAS data in the context of the AS-NHS data

**Fig. 3:**
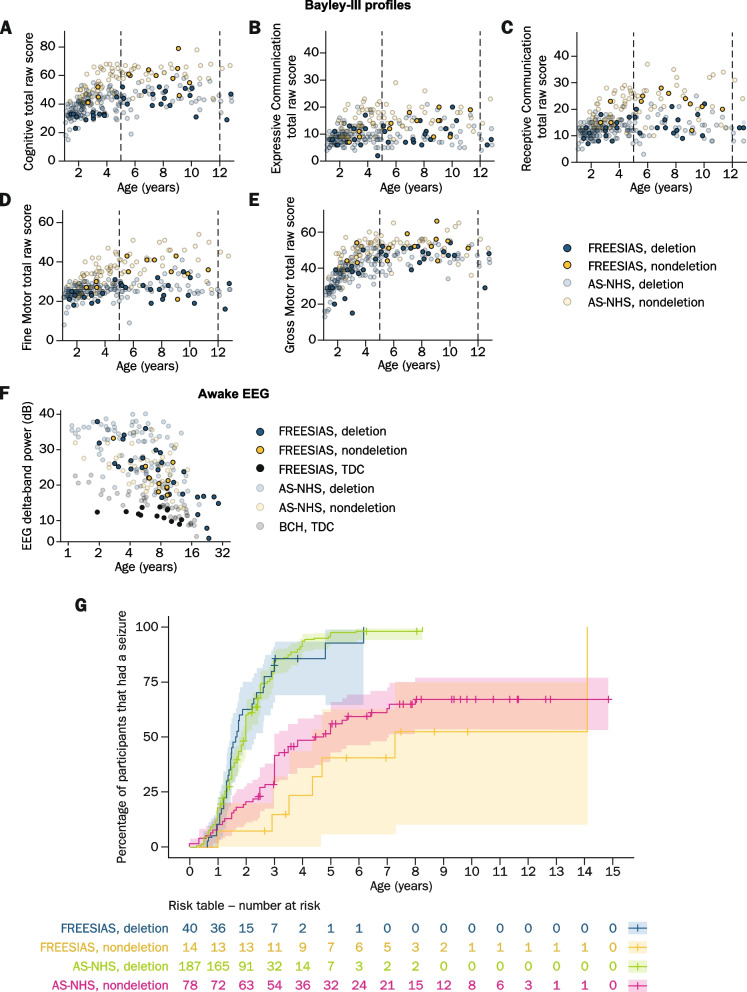
Bayley-III profiles obtained using FREESIAS and AS-NHS data. Mean Bayley-III raw scores in participants with AS deletion and nondeletion aged 1–12 years, from FREESIAS and the AS-NHS: **A** Cognitive domain, **B** Expressive Communication domain, **C** Receptive Communication domain, **D** Fine Motor domain, **E** Gross Motor domain. **F** EEG data in participants with AS deletion, nondeletion, and TDC from FREESIAS, AS-NHS, and BCH. Axes are plotted on logarithmic scales. **G** Kaplan–Meyer analysis of cumulative seizure data, stratified by underlying genotype and data source, in participants with AS deletion and nondeletion from FREESIAS and the AS-NHS. The table indicates the number of participants at risk for each group at 12-month intervals. One FREESIAS participant that presented their first seizure at over 18 years old was excluded from this analysis in order to allow for a direct comparison to the AS-NHS analysis that included pediatric population exclusively. *AS* Angelman syndrome, *AS-NHS* Angelman Syndrome Natural History Study, *Bayley-III* Bayley Scales of Infant and Toddler Development® – Third Edition, *BCH* Boston Children’s Hospital, *EEG* Electroencephalogram, *TDC* Typically developing children

Comparison of raw scores of Bayley-III baseline data collected in FREESIAS and the AS-NHS [44] was performed only on the 1–12 years age range due to limited data availability on adult individuals with AS in the AS-NHS (Figs. 3A–E; Table S5). For FREESIAS versus AS-NHS data, the mean (± SD) raw score for the Cognitive domain in participants with AS aged 1–4 years was 35.5 (± 7.4) vs. 41.5 (± 10.3) and 49.9 (± 11.1) vs. 51.1 (± 11.4) for those aged 5–12 years. The mean (± SD) raw score for the Receptive Communication domain was 12.9 (± 4.2) vs. 13.8 (± 4.5) in participants with AS aged 1–4 years and 17.4 (± 5.6) vs. 17.6 (± 7.6) for those aged 5–12 years. For the Expressive Communication domain, the mean (± SD) raw score was 9.0 (± 3.7) vs. 10.4 (± 3.8) in participants with AS aged 1–4 years and 11.9 (± 4.3) vs. 12.7 (± 5.2) for those aged 5–12 years. The mean (± SD) raw score for the Fine Motor domain was 24.1 (± 3.8) vs. 26.6 (± 5.4) in participants with AS aged 1–4 years and 29.6 (± 7.3) vs. 32.5 (± 8.8) for those aged 5–12 years, while for the Gross Motor domain, mean (± SD) raw scores were 36.6 (± 10.7) vs. 38.1 (± 9.4) in participants with AS aged 1–4 years and 49.3 (± 6.7) vs. 48.8 (± 6.6) for those aged 5–12 years (Table S5). A time-to-event analysis was performed to evaluate seizure onset age in participants with AS, and the results were compared with the AS-NHS data [26, 44] (see Fig. 3G). Results from the FREESIAS study indicated that participants with deletion AS have an earlier seizure onset and a higher seizure prevalence overall, consistent with previous findings from the AS-NHS study [26, 44].

Delta power derived from 10 min of awake EEG recorded in the afternoon of the first day was quantified for baseline recordings. Excess EEG delta power was detected for participants with AS compared with TDC, in line with previous reports (see Fig. 3F). The data also quantitatively matched previous ~ 30-min awake state recordings in participants with AS, thus confirming that usable EEG data were collected in the home setting in the present study [16, 17].

### Study expectations and feedback

**Fig. 4:**
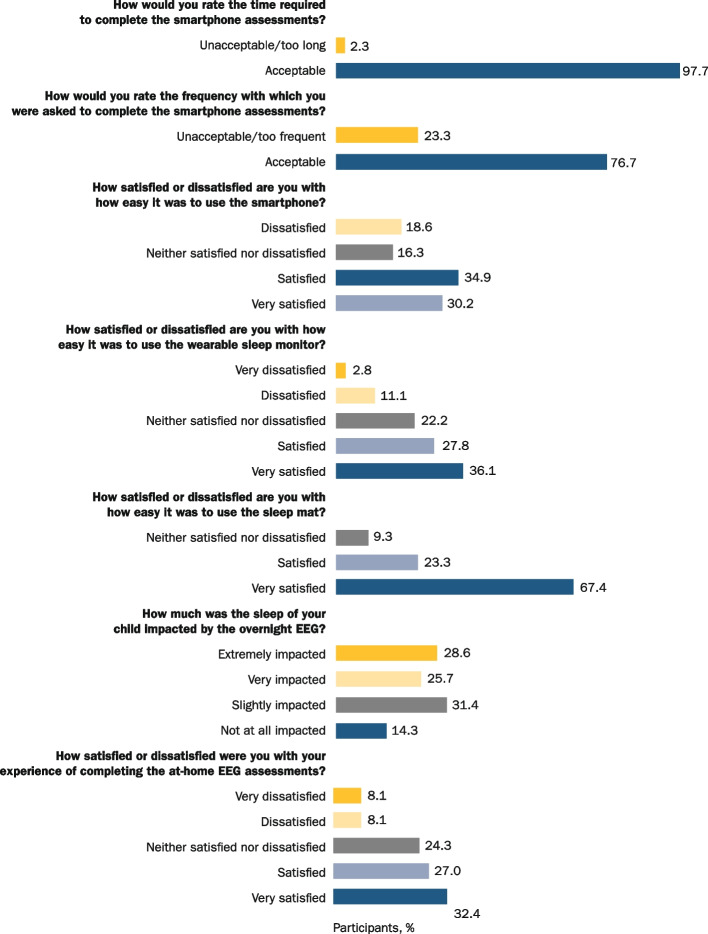
Study expectations and output from feedback questionnaires. *EEG* electroencephalogram

Insights from caregivers of individuals with AS (*N* = 54) were obtained from multiple choice feedback questionnaires (Fig. 4; Table S6). Understanding the motivation of caregivers of individuals with AS to participate in a nondrug observational study supports the design and execution of future clinical trials. Based on multiple choice feedback questionnaires collected from these caregivers, key motivators for choosing to participate in the study included: contributing to medical research (93%; 50/54); getting more treatment options (82%; 44/54); gaining a better understanding of AS (76%; 41/54); and raising awareness of AS in the community (41%; 22/54). In caregivers of TDC (*N* = 20), the main motivating factors to participate in the study included contributing to medical research (95%; 19/20) and getting more treatment options for individuals with AS (60%; 12/20).

In total, 74 caregivers completed the questionnaire about the in-clinic visits, including 54 caregivers of individuals with AS and 20 of TDC, although the number of caregivers responding to different parts of the questionnaire differed depending on the question, their previous involvement in clinical research, and willingness to answer. Most of the caregivers of individuals with AS (86%; 37/43) and all of those of TDC (100%; 17/17) expressed that they were satisfied or very satisfied with the experiences of those participants in completing the in-clinic assessments. Most of the caregivers of individuals with AS also deemed the duration of clinic visits for the study to be acceptable (79%; 34/43) and were satisfied or very satisfied (95%; 41/43) with the support given during the clinic visits. Importantly, due to the large number of assessments at the clinical visits, caregivers could choose to perform the assessments over 2 days, which was preferred by a third of all caregivers (35%; 21/60).

In total, 50 caregivers reported on their at-home visit experiences, including 38 caregivers of individuals with AS and 12 of TDC. Home visits were deemed to be a slight (45%; 17/38) or moderate (29%; 11/38) burden.

When reporting on the ease of smartphone use, 28/43 (65%) of the caregivers of individuals with AS were satisfied or very satisfied with it, 33/43 (77%) rated the frequency of smartphone assessments as acceptable, and 42/43 (98%) deemed the time required to complete these assessments to be acceptable. Further answers to the smartphone part of the questionnaire revealed that 59% (22/37) of the respondent caregivers of individuals with AS were very satisfied or satisfied with the experience of the at-home EEG assessment. This may relate to the percentage of caregivers who found the sleep of the child slightly impacted (31%; 11/35) or very/extremely impacted (54%; 19/35). Also, in contrast to the aforementioned slight/moderate burden reported for at-home visits, caregivers of individuals with AS who commented on their preference for at-home vs. in-clinic visits. (*n* = 40) largely preferred home as the future visit setting (75%; 30/40).

When reporting on the at-home sleep devices, 91% (39/43) of the respondent caregivers of individuals with AS were very satisfied or satisfied with the sleep mat; the score was higher than the actigraph, for which 64% (23/36) of the respondent caregivers of individuals with AS reported that they were satisfied or very satisfied.

In total, 79% (34/43) of caregivers of individuals with AS who commented on their overall experience of the study were satisfied or very satisfied with their experience, with only 9% (4/43) being dissatisfied or very dissatisfied. The main reasons stated for this dissatisfaction included technical issues with some of the DHTs and the request to use digital rather than paper assessments.

## Discussion

### Feasibility and adherence for clinical outcome assessments and digital health technologies in FREESIAS

In line with the primary objective, this prospective FREESIAS trial demonstrated the feasibility and acceptability of conducting assessments in clinic and at home in participants with AS. The overall adherence results indicate that the key clinical aspects of AS identified by caregivers and clinicians —seizures, sleep, motor function, expressive communication, cognition, self-care, and maladaptive behaviors — can be measured through COAs and DHTs [11, 14]. Adherence and/or uptake was likely negatively impacted by the COVID-19 pandemic and fatigue resulting from the use of certain DHTs. For example, lower adherence for the Bayley-III was observed during Clinic Visit 2 as fewer in-clinic visits occurred due to the COVID-19 pandemic; thus, this may underestimate the Bayley-III adherence in future clinical trials.

The COVID-19 pandemic began approximately 6 months after the start of the trial, which restricted travel and in-person interactions, limited the number of clinic and home visits, and decreased enrollment pace. Amending the protocol to allow for remote COAs enabled adherence to remain high, showcasing investigational site flexibility and successful implementation of a decentralized study setup. However, the resultant reduction in home visits, to less than 50% of those originally planned, proportionately reduced the amount of EEG recordings obtained. Notably, this study illustrates the acceptance and importance of telehealth generated by necessity for remote solutions during the pandemic. Such solutions allowed the participants and their caregivers to continue the data collection while potentially minimizing the risk of them contracting COVID-19. However, for future global clinical trials, telehealth might face local or regional challenges due to absence of supportive IT infrastructure, limited experience using the technologies, or cultural acceptance, hence requiring further feasibility and acceptability assessment [51].

General characteristics relating to medical history, as well as the proportion of participants with deletion and nondeletion AS, were similar to those previously published [52] including the AS-NHS, suggesting that the study was representative of the AS population. Notably, the relatively small sample size of each tested group and the exploratory nature of the observational study objectives deemed any statistical testing inappropriate (Table 1). However, some imbalance can be seen when observing the frequency distribution of deletion vs. nondeletion individuals in the three age subgroups (i.e., deletion in 81% of the 1–4 years group, 63% of the 5–12 years group, and 83% of the ≥ 18 years group), expectedly deeming this a random finding.

Furthermore, consistently with the more severe clinical phenotype previously described [21–25], participants with deletion AS consistently showed lower scores, i.e., more impairment in COAs compared with those with nondeletion AS. In addition, the cross-sectional changes of scores with age were consistent with previously published data [29]. The similarities in results between the FREESIAS and the AS-NHS studies further support the appropriateness of combining the findings to increase the amount of available data, improve the understanding of AS, and support AS clinical trial designs.

### Challenges associated with clinical outcome assessments and digital health technologies identified in FREESIAS

No publicly available AS-specific CGI-S scale existed at the start of the study. Therefore, a single question CGI scale was used to characterize the severity of the condition of participants with AS, which lacked a prespecified anchor to ensure intra- and inter-rater reliability. Although participants with deletion AS were more frequently rated as “*Markedly ill”* (score of 5) and “*Severely ill*” (score of 6) compared with participants with nondeletion AS, overall, the CGI-S showed little distinction between AS genotypic subgroups. This is potentially due to the heterogeneous nature of AS symptoms but is more likely driven by the aforementioned limitations and suggests that the 1-item scale is not appropriate for future clinical studies in the AS population. An adapted CGI-S that assesses each functional domain, with clearly defined anchors, is likely to be more useful.

For the seizure diary, the absence of an option for caregivers to report if no seizures occurred made it difficult to distinguish between poor adherence versus true absence of seizures; therefore, regular confirmation of absence of seizures should be implemented in future seizure diaries.

Seizure frequency reporting as part of the seizure history at baseline could also be improved in future clinical trials. The categories provided for frequency reporting may have been insufficient for this population. For example, a participant with 385 seizures in 463 days logged in the diaries will report the same frequency category during the clinical visit as a participant with 17 seizures in 360 days, i.e., “ < 1 seizure/day”. More fine-grained seizure frequency bins or reporting the actual number of seizures over an observation period, should be considered. Identifying improved categories on seizure frequency tailored to the AS population, would likely improve overall data quality. A key challenge identified in this study pertained to the acceptance and adherence to DHTs, as well as potential technologic barriers. The feasibility of using digital measures at home is dependent upon access to technology, which potentially biased the inclusion of participants to those who were expected to be largely able to continuously adhere to the study requirements. Providing participants with DHTs, telecommunications technologies, and technical support throughout the study by the Sponsor should be considered as part of the study feasibility process and would ensure that participants are not excluded or discouraged from taking part if they do not have their own technology.

### Multidimensional sleep analysis in FREESIAS and at-home overnight electroencephalography

Sleeping difficulties are common in the majority of individuals with AS, which in turn impacts the sleep of their caregivers/families [53]. Sleep behavior of study participants and their caregivers was assessed through standardized questionnaires, a sleep diary, sleep actigraphy, and a sleep mat. The multidimensional approach allowed for comparison of newer DHTs (e.g., sleep mat and actigraph) against more established COAs such as the PSQI and SNAKE. Future comparison between the PSQI, CSDI and ESS, the SNAKE, and HOST may help to determine which COA is the most suitable for future clinical trials.

Adherence for the sleep diary was only considered acceptable, despite high caregiver satisfaction. However, the substantial number of missing inputs from caregivers might pose a challenge to data analysis. Implementing shorter diary completion windows at regular intervals might reduce burden, and in turn, increase adherence and data quality. The sleep mat offered low participant burden and high caregiver satisfaction with similar adherence to the sleep diary and therefore is a viable DHT for use in studies including participants with AS. In comparison, monitoring sleep using an actigraph was more challenging due to lower levels of adherence. To reduce burden, participants were asked to wear the actigraph over the 11-day monitoring windows during sleep only, instead of the 24/7 regimen recommended by the manufacturer, and were given a free choice on wearing location. Despite these adjustments, caregivers of both participants with AS and TDC found the actigraph less convenient than the sleep mat, suggesting that the sleep mat might be more suitable for long-term sleep monitoring in AS.

This study explored at-home assessment with the goal of reducing burden on participants and their caregivers, including pioneering home visits to perform overnight EEG/limited PSG recordings in a multicenter clinical trial setting. Such recordings can, in principle, support a multitude of analyses including quantitative EEG in the awake and sleep state, analyses of sleep structure and elements, and analyses of epileptiform activity and seizures. EEG recorded in the home setting provided usable data in most cases, which were confirmed by recovering the AS phenotype of excess EEG delta power.

### Novel strategies to support future rare disease clinical trials

The feedback questionnaire data provided valuable insight into the key motivations for families to join observational studies and assessed the perceived burden of participating in clinical studies. While most caregivers were satisfied with their overall study experience, perceived burdens included technical difficulties, intolerance to assessment, and impact on sleep in participants with AS. However, most caregivers indicated that they would still prefer at-home EEG over in-clinic EEG assessment. The novel insights gained here will collectively support optimization of the design and execution of future clinical trials. Furthermore, these insights also support the use of the novel study-specific seizure diary and sleep diary as well as the application of the customized approach to the limited implementation of PSG in the home setting, with both appearing to be feasible for severely cognitively and behaviorally challenged populations across the age span. These elements can be refined further and implemented more widely to improve quality of data and insights in these domains.

This study represents a precompetitive collaboration between industry, academia, and patient advocacy groups to drive study design and study implementation in AS, with the goal of obtaining valuable information for all parties involved and reducing overall burden for families. Furthermore, the FREESIAS study was amended to provide participants with AS with priority screening for future AS clinical drug trials sponsored by the funding industry partners, upon completing at least 6 months in the FREESIAS study. Based on investigator feedback and the increase in enrollment after publicly announcing this approach, it appeared to be a strong motivating factor for caregivers’ participation and may be a strategy that would benefit future clinical trials in rare diseases with limited patient populations and provide an extended baseline for the subsequent drug trial. With over a third of participants with AS co-enrolled in the AS-NHS, sharing data from overlapping COAs between the FREESIAS and AS-NHS studies was agreed in order to reduce the burden on study participants and their caregivers, and to avoid negatively impacting the ongoing long-term AS-NHS observational study.

## Conclusions

This study involved both in-clinic and at-home observations with many moving parts during the COVID-19 pandemic, but despite such challenges generated valuable insights into relevant COAs and DHTs to measure key aspects of AS. Among them, it demonstrated that while participants were highly adherent to the prescribed COAs, the DHTs remain variedly popular. The detailed questionnaires, however, highlighted a generally high acceptance of the employed techniques, and showed that most participants were either satisfied or very satisfied with their overall experience of the study.

The results presented herein pose questions to be addressed through advanced evaluations that would, for example, validate newer COAs through comparing them with older COAs. Such additional longitudinal analyses of these data are already planned and shall be published once ready. Taken together, the current results and future works derived from them may inform the design and strengthen the analysis of future clinical trials in AS and other neurological and neurodevelopmental conditions and rare diseases.

## Supplementary Information

**Additional file 1.**

## Data Availability

For up-to-date details on Roche's Global Policy on Sharing of Clinical Study Information and how to request access to related clinical study documents, see here: https://go.roche.com/datasharing. The datasets generated and/or analyzed during the current study are not publicly available, but anonymized data may be made available in an appropriately modified format to qualified investigators upon reasonable request. Anonymized records for individual patients across more than one data source external to Roche cannot, and should not, be linked due to a potential increase in risk of patient re-identification.

## Abbreviations

ABC-2-C: Aberrant Behavior Checklist Second Edition – Community Version
AS: Angelman syndrome
AS-NHS: Angelman Syndrome Natural History Study
Bayley-III: Bayley Scales of Infant and Toddler Development® – Third Edition
CGI-S: Clinical Global Impression – Severity
ClinRO: Clinician-reported outcomes
COA: Clinical outcome assessment
COVID-19: Coronavirus disease 2019
CSDI: Composite Sleep Disturbance Index
DHT: Digital health technology
EEG: Electroencephalography
EMG: Electromyography
ESS: Epworth Sleepiness Scale
EQ-5D-5L: European Quality of Life 5-Dimensions Questionnaire-Five Levels
EQ-5D-Y: European Quality of Life 5-Dimensions Questionnaire-Youth
FMS: Functional Mobility Scale
FREESIAS: FiRst Endpoint-Enabling Study in Angelman Syndrome
HOST: Holistic assessment of sleep and daily troubles in parents of children with severe psychomotor impairment
ID: Imprinting defects
IQR: Interquartile range
ObsRO: Observer-reported outcomes
PedsQL™ 4.0 Core: Pediatric Quality of Life Inventory™ Generic Core Scales, Version 4.0
PedsQL™-FIM: Pediatric Quality of Life Inventory™ Family Impact Module
PerfO: Performance outcome
PSG: Polysomnography
PSQI: Pittsburgh Sleep Quality Index
SD: Standard deviation
SNAKE: Schlaffragebogen für Kinder mit Neurologischen und Anderen Komplexen Erkrankungen (Sleep Questionnaire for Children with Severe Psychomotor Impairment)
TDC: Typically developing children
UBE3A: Ubiquitin-protein ligase E3A
UPD: Uniparental disomy
Vineland-3: Vineland Adaptive Behavior Scales® – Third Edition
