# Supplementary material for: Enabling endpoint development for interventional clinical trials in individuals with Angelman syndrome: a prospective, longitudinal, observational clinical study (FREESIAS)
Source: J Neurodev Disord. 2023 Jul 26;15:22. doi: 10.1186/s11689-023-09494-w (PMC10373389; doi:10.1186/s11689-023-09494-w)
Supplement: Supplementary file 1 — Additional file 1. [file 11689_2023_9494_MOESM1_ESM.docx]

**Supplement to: Enabling endpoint development for interventional clinical trials in individuals with Angelman syndrome: a prospective, longitudinal, observational clinical study (FREESIAS)**

Jorrit Tjeertes, Carlos A. Bacino, Terry Jo Bichell, Lynne M. Bird, Mariana Bustamante, Rebecca Crean, Shafali Jeste, Robert W. Komorowski, Michelle L. Krishnan, Meghan T. Miller, David Nobbs, Cesar Ochoa-Lubinoff, Kimberly Parkerson, Alexander Rotenberg, Anjali Sadhwani, Mark D. Shen, Lisa Squassante, Wen-Hann Tan, Brenda Vincenzi, Anne C. Wheeler, Joerg F. Hipp and Elizabeth Berry-Kravis

Contents

[Additional methods 2](#_Toc117862758)

[Schedule of activities 2](#_Toc117862759)

[Inclusion criteria 5](#_Toc117862760)

[Exclusion criteria 5](#_Toc117862761)

[Objectives and endpoints 6](#_Toc117862762)

[Clinical outcome assessments (COAs) 8](#_Toc117862763)

[Digital health technologies (DHTs) 18](#_Toc117862764)

[Mapping of COAs and DHTs to AS symptomatic domains 21](#_Toc117862765)

[Supplementary references 24](#_Toc117862766)

[Supplementary tables and figures 27](#_Toc117862767)

[Supplementary figures 39](#_Toc117862768)

# Additional methods

## Schedule of activities

| **Item** | **Applicable to TDC** | **Pre-screening** | **Clinic Visit 1** | **Home  Visit 1** | **Home  Visit 2** | **Home  Visit 3** | **Unscheduled clinic visit** | **Early Withdrawal/ Clinic Visit 2** |
| --- | --- | --- | --- | --- | --- | --- | --- | --- |
| **Time** |  |  | **0** | **Up to 8 weeks after  Clinic Visit 1** | **3 months after Home Visit 1 +/- 3 weeks** | **6 weeks or less before Clinic Visit 2** | **As needed ^(n)^** | **Any time/  12 months after  Clinic Visit 1**  **+/- 2 weeks** |
| **Activities concerning participants** ^(e)^ |  |  |  |  |  |  |  |  |
| Clinical and/or molecular diagnosis of AS from medical record | no | X^(a)^ | X^(b)^ |  |  |  |  |  |
| Written informed consent  and assent | yes |  | X^(b)^ |  |  |  |  |  |
| Molecular diagnosis of AS (from medical record) | no |  | X |  |  |  |  |  |
| Blood sample for full molecular diagnosis of AS (if medical record is not sufficient) | no |  | X^(d, o)^ |  |  |  |  |  |
| Blood sample for RBR (optional) | no |  | X |  |  |  |  |  |
| Medical and medication history, diet | yes |  | X |  |  |  |  |  |
| Demographics/social status | yes |  | X |  |  |  |  |  |
| Physical examination (including neurologic examination) | yes |  | X |  |  |  |  | X |
| Height^(c)^ | yes |  | X |  |  |  |  | X |
| Weight | yes |  | X |  |  |  |  | X |
| Head circumference | yes |  | X |  |  |  |  | X |
| Tanner staging | yes |  | X |  |  |  |  | X |
| Hand-over devices and training | yes |  | X |  |  |  |  |  |
| Bayley-III | no |  | X |  |  |  |  | X |
| Functional Mobility Scale (FMS) | yes |  | X |  |  |  |  | X |
| Clinical Global Impression – Severity (CGI-S) | no |  | X |  |  |  |  |  |
| Clinical Global Impression – Change (CGI-C) | no |  |  |  |  |  |  | X |
| Vineland-3 | yes |  | X^(f)^ |  |  |  |  | X^(f)^ |
| Communication Matrix (parent/caregiver) | yes |  | X |  |  |  |  | X |
| Communication Matrix (school) | yes |  |  | once | | |  |  |
| At-home sleep assessment (limited PSG) | yes |  |  | X^(g)^ | X^(g)^ | X^(g)^ |  |  |
| Awake EEG | yes |  |  | X^(g)^ | X^(g)^ | X^(g)^ |  |  |
| Sleep actigraphy (if feasible) | yes |  |  | X^(h)^ |  |  |  |  |
| Sleep mat | yes |  |  | continuous^(i)^ | | |  |  |
| SNAKE | no |  | X^(j)^ |  |  |  |  | X^(j)^ |
| CSDI | no |  | X^(j)^ |  |  |  |  | X^(j)^ |
| ABC-2-C | no |  | X^(j)^ |  |  |  |  | X^(j)^ |
| PedsQL™ 4.0 Core Proxy | no |  | X^(j)^ |  |  |  |  | X^(j)^ |
| EQ-5D-Y Proxy | no |  | X^(j)^ |  |  |  |  | X^(j)^ |
| Sleep and nap diaries | yes |  |  | at most daily via smartphone app^(i)^ | | |  |  |
| Seizure diary | no |  |  | daily via smartphone app^(i)^ | | |  |  |
| Adverse events | yes | continuous | | | | | | |
| Concomitant medication and changes in diet | yes | continuous | | | | | | |
| Contact caregivers for updates | yes |  |  | approximately every 6 to 8 weeks^(m)^ | | |  |  |
| **Activities concerning parents/caregivers** |  |  |  |  |  |  |  |  |
| Written informed consent | yes |  | X |  |  |  |  |  |
| Sleep survey (HOST) | no |  | X^(j)^ |  |  |  |  | X^(j)^ |
| PSQI | no |  | X^(j)^ |  |  |  |  | X^(j)^ |
| ESS | no |  | X^(j)^ |  |  |  |  | X^(j)^ |
| PedsQL™-FIM | no |  | X^(j)^ |  |  |  |  | X^(j)^ |
| EQ-5D-5L | no |  | X^(j)^ |  |  |  |  | X^(j)^ |
| Sleep diary | yes |  |  | at most daily via smartphone app^(i)^ | | |  |  |
| Study expectations | yes |  | X^(k)^ |  |  |  |  |  |
| Study feedback | yes |  |  |  |  |  |  | X^(l)^ |
| (a) Prescreening: study coordinator to liaise with Investigator to identify potentially suitable patients with clinically confirmed and/or molecularly confirmed diagnosis of AS from clinic patient records.  (b) Visit 1: after positive prescreening, formal screening will be performed on the day of Clinic Visit 1. Checking of in/exclusion criteria, clinical assessments, and data collection will only start post-signing of the Informed Consent Form.  (c) Ulnar length may be used as a proxy of height if standing height measurement not possible or impacted by clinical factors.  (d) Blood sample for molecular diagnosis of AS to be taken from participants with AS only and only if the available information from medical records is not sufficient to determine the genetic etiology of AS (deletion versus nondeletion). If the sample is missed, it will be collected at the next scheduled clinic visit.  (e) At the discretion of the Sponsor, any assessments described in Section 8 that were performed as part of another nondrug study within the past 3 months can be used across both studies and may not need to be repeated for this study.  (f) Parents/caregivers may choose to complete the Vineland questionnaire at the clinical site or remotely, e.g., via the phone or Skype, from home.  (g) Home visit: will be performed by trained personnel and will include awake EEG and limited PSG. Order of assessments (start of assessments in the afternoon): awake EEG, PSG, and awake EEG. Each of the three home visits consist of at least one but up to 3 nights. If repeat measurements on consecutive days are not feasible, the 2nd and 3rd measurement can be scheduled up to 2 weeks after the previous measurement at a date agreed with the parent/caregiver. Parents/caregivers must agree to at least one home visit with at least one attempt for an EEG and/or limited PSG.  (h) Sleep actigraphy data will be collected starting ≤10 nights before the first and second at-home sleep assessment and continue during all nights of the first and second at-home sleep assessment. If wearing the actigraphy device is significantly impacting sleep, the assessment can be stopped.  (i) Sleep mat and smartphone app to be used at home between Clinic Visit 1 and Clinic Visit 2 or Early Withdrawal Visit.  (j) Parents/caregivers may choose to complete the assessment at home after demonstration in-clinic up to 4 weeks after the first clinic visit and within the 4 weeks before the second clinic visit.  (k) Study personnel to record parent/caregiver study expectations as free-text entry in the eCRF at Clinic Visit 1 post-signing of the Informed Consent Form.  (l) Parent/caregiver to complete satisfaction questionnaire (feedback) at home before the last visit or early withdrawal from the study.  (m) Study personnel to contact parents/caregivers to follow up on at least the following: AEs, changes in concomitant medications and diet, and compliance with sleep, nap, and seizure diaries.  (n) Unscheduled clinic visit to follow up on AEs as needed and in case reconsent is required.  (o) For very rare cases, a parental blood sample(s) is required to obtain a full molecular diagnosis. A specific consent for this is provided in the Informed Consent Form.  *ABC-2-C* Aberrant Behavior Checklist Second Edition – Community Version, *AE* adverse event, *AS* Angelman syndrome, *Bayley-III* Bayley Scales of Infant and Toddler Development® – Third Edition, *CGI-C* Clinical Global Impression – Change, *CGI-S* Clinical Global Impression – Severity, *CSDI* Composite Sleep Disturbance Index, *eCRF* electronic case report form, *EEG* electroencephalogram, *EQ-5D-5L* European Quality of Life 5-Dimensions Questionnaire-Five Levels, *EQ-5D-Y* European Quality of Life 5-Dimensions Questionnaire-Youth, *ESS* Epworth Sleepiness Scale, *FMS* Functional Mobility Scale, *HOST* holistic assessment of sleep and daily troubles in parents of children with severe psychomotor impairment, *PedsQL™ 4.0 Core* Pediatric Quality of Life Inventory™ Generic Core Scales, Version 4.0, *PedsQL™-FIM* Pediatric Quality of Life Inventory™ Family Impact Module, *PSG* polysomnography, *PSQI* Pittsburgh Sleep Quality Index, *RBR* Research Biosample Repository, *SNAKE* Schlaffragebogen für Kinder mit Neurologischen und Anderen Komplexen Erkrankungen (Sleep Questionnaire for Children with Severe Psychomotor Impairment), *TDC* typically developing children, *Vineland-3* Vineland Adaptive Behavior Scales® – Third Edition | | | | | | | | |

## Inclusion criteria

Participants are eligible to be included in the study only if all the following criteria apply:

- Parent or legal guardian/representative (caregiver) willing to give written informed consent and to comply with study requirements
- Availability of parent or other reliable and consistent caregiver who agrees to accompany the participant to all clinic visits to provide information about the participant’s behavior and symptoms
- **Age**
  - Participants with Angelman syndrome (AS): participant must be ≥1 to ≤12 years of age or ≥18 years of age at the time of signing of the informed consent by the parent/caregiver
  - Typically developing children (TDC): participant must be ≥1 to ≤12 years of age at the time the parent/caregiver signs the Informed Consent Form
- **Type of participants and condition characteristics**
  - Participants with AS: clinical and full or partial molecular diagnosis of AS at the time the parent/caregiver signs the Informed Consent Form
  - Participants with partial molecular diagnosis of AS: if only partial diagnosis is available and this partial molecular diagnosis is not sufficient to determine the genetic etiology of AS, parent/caregiver agrees to have a blood sample taken from their child for a more detailed molecular diagnosis
  - Note: the sample will be taken at Visit 1; availability of the result is not a pre-requisite for inclusion
- **Sex**
  - Male and female participants
- **Participation in other studies**
  - At the discretion of the Sponsor, participants with AS may also enroll into other nondrug observational studies

## Exclusion criteria

Participants are excluded from the study if any of the following criteria apply:

- **Medical conditions**
  - Any condition that may significantly interfere with the assessment of AS and that is clearly not related to this condition
- **Prior/concurrent clinical study experience**
  - Previous participation (within 4 weeks of screening or 5 half-lives of the investigational drug, whichever is longer)
  - Current or planned participation (within the study duration) in any investigational drug, biologic agent, or device study
  - Previous participation in a gene therapy or gene editing study
- **Enrollment restriction** (only one AS patient and one TDC per family can enroll in the study)
  - Participants with AS: a sibling with AS is already participating in this study
  - TDCs: a sibling without AS is already participating in this study
- **Other**
  - Parent/caregiver not willing to comply with study requirements

## Objectives and endpoints

| **Primary objective** | |
| --- | --- |
| To evaluate the feasibility and value of measures acquired in-clinic and at home and to inform their use in possible future multicenter multinational clinical efficacy trials in individuals with AS | |
| **Secondary objectives** | **Endpoints** |
| To characterize features of sleep in patients with AS | Sleep structure measures derived from sleep staging of limited polysomnography (PSG) recording including: duration of the longest uninterrupted sleep episode per night; total sleep time; time in bed; sleep latency; duration of time awake after sleep onset; number of awakenings; duration and percentage of REM and non-REM sleep; fraction of REM sleep  Sleep spindles (frequency, amplitude, duration) |
| To characterize seizures and electroencephalogram (EEG) in patients with AS | Seizures (frequency and type of seizures)  Epileptiform abnormalities (rate of epileptiform spikes)  Spectral EEG features (delta power) |
| To characterize neurocognitive functions and adaptive behavior in patients with AS | Bayley Scales of Infant and Toddler Development^®^ – Third Edition (Bayley-III): Cognitive, Language, Motor, Social–Emotional, and Adaptive Behavior Scales and Subscales  Vineland Adaptive Behavior Scales^®^ – Third Edition (Vineland-3): Adaptive Behavior Composite Score and Socialization, Communication, Daily Living Skills, and Motor Skills Domain Scores  Communication Matrix |
| **Tertiary/exploratory objectives** | **Endpoints** |
| To characterize features of sleep in AS using remote monitoring | Sleep features derived from sleep actigraphy, sleep mat, and sleep and nap diaries including sleep onset latency, number of awakenings, duration of wake time after sleep onset, total sleep time, and sleep efficiency |
| To assess aberrant/maladaptive behaviors | Aberrant Behavior Checklist Second Edition – Community Version (ABC-2-C) |
| To assess global clinical status severity and change from baseline | Clinical Global Impression – Severity (CGI-S) score  Clinical Global Impression – Change (CGI-C) score |
| To investigate differences in clinical features of individuals with AS with different genetic architecture, and between individuals with AS and TDC | Sleep and EEG measures as described above |
| To assess mobility in individuals with AS | Functional Mobility Scale (FMS) |
| To assess health-related quality of life in individuals with AS as reported by parent/caregiver | Pediatric Quality of Life Inventory™ Generic Core Scales, Version 4.0 (PedsQL™ 4.0) Core Proxy score  European Quality of Life 5-Dimensions Questionnaire-Youth (EQ-5D-Y) Proxy |
| To assess severity of sleep disturbance as reported by parent/caregiver | Schlaffragebogen für Kinder mit Neurologischen und Anderen Komplexen Erkrankungen (SNAKE; Sleep Questionnaire for Children with Severe Psychomotor Impairment) and Composite Sleep Disturbance Index (CSDI) |
| To measure seizure type, frequency severity as reported by parent/caregiver | Seizure diary |
|  |  |
|  |  |
| To assess quality of life in caregivers and families of individuals with AS | Pediatric Quality of Life Inventory™ Family Impact Module (PedsQL™-FIM) score  Holistic assessment of sleep and daily troubles in parents of children with severe psychomotor impairment (HOST) score  Epworth Sleepiness Scale (ESS)  Pittsburgh Sleep Quality Index (PSQI)  European Quality of Life 5-Dimensions Questionnaire-Five Levels (EQ-5D-5L) |
| To ascertain parent/caregiver’s experience of participating in the study | Study expectations  Study feedback questionnaire |

## Clinical outcome assessments (COAs)

### Bayley-III

The Bayley Scales of Infant and Toddler Development^®^ – Third Edition (Bayley-III) is the most extensively used measure of infant and toddler development in clinical and research practice. The Bayley-III were first published in “The Bayley Scales of Infant Development” (1969) [1] and have been used extensively worldwide since then to assess the development of infants [2]. Compared with the previous version, the third edition of the Bayley-III, was improved by updating normative data, strengthening psychometric qualities, and changing some items to make administration and scoring easier and more meaningful [3].

The Bayley-III consists of a core battery of five scales, i.e., Cognitive, Language, Motor, Social–Emotional, and Adaptive Behavior. In this study, only the Cognitive, Language, and Motor scales were administered. In addition, completion of the behavior observation inventory form was required.

The Bayley-III is a standardized developmental assessment for children up to 42 months of age, though it can be administered to individuals with developmental disabilities beyond these normative age ranges when significant global delays are present to estimate a developmental age equivalent based on raw scores [2, 4]. Given that individuals with AS present with substantial developmental delays, this population should be evaluated with measures that are suited for their developmental level rather than chronologic age [2]. Therefore, the Bayley-III is a suitable tool that can provide an estimate of developmental functioning in areas related to cognition, speech and language, and motor skills [2].

To ensure consistent administration of the Bayley-III across an investigational site, a standardized administration protocol was generated by a team of clinicians, comprised of psychologists and speech and language pathologists with extensive experience in assessing individuals with AS and was used for all clinical trial participants, independent of their age. Steps to create standardized accommodations for the Bayley-III for individuals with AS included a review of relative difficulty of all items across all subdomains for individuals with AS who received a Bayley-III either clinically or through the initial Natural History Study. Based on this review, standardized guidelines for determining start points were developed (see below). In addition, all accepted accommodations as outlined in the Bayley-III manual (e.g., support of elbows for those with tremor; providing color contrast for the background for visually oriented items) were reviewed for appropriateness with the AS population. Specific recommendations for how and when to use these accommodations were added to the AS-specific Bayley-III manual used in this study [2]. These recommendations for administrations of the Bayley-III were presented to all investigational site raters during the FREESIAS Investigator Meetings and were maintained throughout the study. This included use of the below starting point guidance for the Cognitive, Language, and Motor scales.

To obtain a consistent starting point procedure for all administrations, parents were asked questions to estimate the current level for each domain, and the person administering the Bayley-III was asked to then start one category *below* the estimated level. The specific guidelines for each of the subscales are listed below.

***Cognitive scale starting point:***

***Receptive scale starting point:***

Start with **Start Point M** and go forward or backward as needed. Give credit for clear eye gaze if individual indicates object rather than just pointing.

***Expressive scale starting point:***

Start at **Start Point A**

***Fine motor scale starting point:***


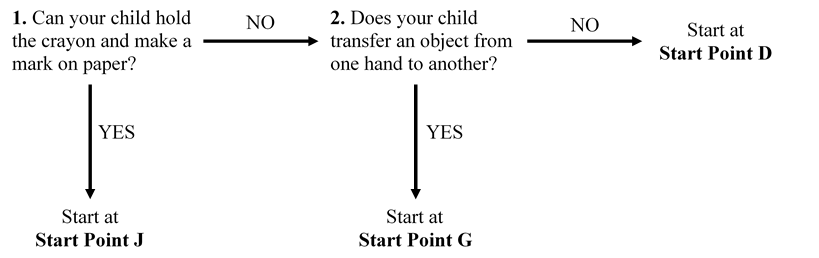


***Gross motor scale starting point:***

If walking independently, start at **Start Point K**

The Bayley-III was used for participants with AS only. Depending on the age of the child it takes between 30 to 90 minutes to complete the scales.

### Vineland-3

The Vineland-3 is an instrument that measures communication, daily living skills, socialization, and maladaptive behavior [5]. The Comprehensive Interview Form (i.e., a semi-structured interview) was administered to the participant’s parent/caregiver: the Investigator or a designee asked the parent/caregiver open-ended questions relating to the participants activities and behavior per standardized Vineland-3 administration procedures. All individuals were administered items starting at item 1 for each of the domains. The Motor domain was administered to all individuals. Of note, the Vineland-3 Expressive Language domain allowed for use of sign language and augmentative or alternative communication (AAC) in its scoring. Standardized domain scores were obtained for the individual domains of Socialization, Communication, Daily Living Skills, and Motor Skills. An adaptive behavior composite score was also generated. Standardized scores on the domains and composite score range from 20 to 160, with higher scores indicating better functioning.

Both the parents/caregivers for individuals with AS and TDC were interviewed. The interview takes approximately 45–60 minutes to complete and can be performed at the clinic or remotely, e.g., via the phone or Skype.

### CGI-S

The Clinical Global Impression – Severity (CGI-S) is a single-item, clinician-rated measure, assessing the clinician’s impression of the severity of a participant’s condition, in this case AS (no other conditions/comorbidities). The CGI-S utilizes a 7-point response scale, ranging from “normal, not at all ill” (1) to “among the most extremely ill subjects” (7). Clinicians make a judgment on a participant’s severity based on the totality of their experience of the population and all information they have available on the individual.

The assessment was used for individuals with AS only and takes about 5 minutes to complete.

### Communication Matrix

As individuals with AS are unlikely to use speech to express themselves, it is important to assess their expressive communication using an instrument that accommodates multimodal responses. The assessment instrument chosen for the current study is the Communication Matrix [6–9].

The Communication Matrix is designed to describe the expressive communication skills of individuals with complex communication needs. It has an online interface [10] that allows assessment of expressive communication in early communicators who use all forms of communication, including speech and AAC. This version is freely available in multiple languages including English and Spanish. The matrix is organized such that individuals who follow a typical course of communication development will progress steadily through seven discrete stages of communication development that typically occur between birth and 2-years-of-age (Level one to Level seven) and will progress through four reasons to communicate from refusing to obtaining, to social interaction, and to information exchange.

The assessment is conducted by answering a series of 24 questions related to the expression of specific messages conveyed. Response options may be scored as not used, emerging, or mastered. Mastered behaviors are defined as actions that are used independently in response to most of the opportunities provided, across contexts and communication partners. Emerging behaviors are defined as actions that are used inconsistently or only when elicited or prompted, in a single context, or with a single communication partner. Once the questions have been answered, the website automatically generates a one-page matrix profile that shows, at a glance, the level of behaviors that the individual uses to communicate, the reasons that he or she communicates and the variety of messages that he or she expresses. The overall Communication Matrix score for each participant can be derived from summing the maximum score (0 = not used, 1 = emerging, 2 = mastered) for each of the 80 unique combinations of levels and messages covered by the assessment, with a maximum score of 160. The assessment can be completed by caregivers, family members, or professionals. Previous research suggests that there may be differences in communication scores as captured by caregivers versus educational or speech–language professionals. The Communication Matrix has several benefits: (a) it assesses expressive communication at the developmental level commonly reported for individuals with AS; (b) it reliably measures expressive communication skills without requiring a spoken response; (c) it provides comprehensive information regarding the entire scope of expressive communication skills, including specific gestures and eight types of alternative symbolic modes; and (d) the Communication Matrix database includes data from professional respondents (i.e., educators, speech language pathologists, and other professionals) on a large sample of individuals with AS, and has been validated in this population [11].

The Communication Matrix was administered to the parents of participants with AS as well as TDC by a skilled clinician and takes approximately 30 minutes to complete. The caregiver/educational professional receives a one-page report that shows the highest “level” of communication that the individual uses to achieve each of those needs.

### SNAKE

The Schlaffragebogen für Kinder mit Neurologischen und Anderen Komplexen Erkrankungen (SNAKE; Sleep Questionnaire for Children with Severe Psychomotor Impairment) questionnaire [12] is a proxy observation instrument for children and adolescents with neurologic and other complex conditions, and can be filled out by those close to the children such as parents or caregivers. SNAKE is validated for ages 1–25 years and is suited to measuring the status and course of therapy. The questionnaire evaluates symptoms and consequences of sleep disturbances, as well as conditions that are known to have direct or indirect impact on sleep in children with severe psychomotor impairment (SPMI). The questionnaire considers the individual’s impaired or limited perception, intellectual ability, limited behavioral repertoire, motor impairment due to underlying condition, and the environmental impact of the condition that makes it less conducive for sleep (e.g., nursing care and artificial ventilation). SNAKE is based on expert opinion and consultation with parents, and the questionnaire can be filled out by the parents or other caregivers of the child without further instructions. Instructions for completion of the questionnaire are at the beginning of each section.

The SNAKE questionnaire contains 54 items (31 measuring sleep conditions and quality; 23 items measuring symptoms and consequences of sleep deficiency). A higher SNAKE score on a particular scale corresponds to more problematic sleep or sleep-related behavior as reported by the caregiver, and an evaluation tool can be used for the calculation of scales, with distribution parameters from a published German normative sample.

The questionnaire was provided to the parents/caregivers of participants with AS only. It takes approximately 15 to 20 minutes to complete. Parents/caregivers could choose to complete the assessment at home after demonstration in the clinic. When it was decided that the assessment would be completed at home, it was required to be finalized up to 4 weeks after Clinic Visit 1 and within the 4 weeks before Clinic Visit 2.

### HOST

The holistic assessment of sleep and daily troubles in parents of children with severe psychomotor impairment (HOST) questionnaire was designed to evaluate the effects experienced by parents or caregivers of children with SPMI-related sleep disturbances [13]. The HOST is completed by the parents/caregivers who answers questions regarding their own sleep problems and the disruption of their lives resulting from their child’s sleep problems during the preceding 4 weeks. The HOST maps the parents’ nighttime disruption due to the amount of time required to care for their child. In addition, the HOST asks for data regarding family changes and several problems (i.e., partnership, social contacts, effect on job performance) resulting from the sleep disturbance of the parent/caregiver, or the child.

The HOST questionnaire contains 12 sets of questions (total 54 items). The sets represent five components of parental sleep behavior: (1) sleep conditions, (2) sleep onset latency, (3) length of sleep, (4) sleep efficiency, and (5) effects of child’s sleep disorders on parents. Components one to three record information about the sleep behavior of parents based on individual items. For Component four (sleep efficiency), a score is calculated from the individual items. Component five records the effects of the sleep disorders of children and adolescents with complex neurologic conditions on their parents in four scales (Scale 1, sleep disturbances; Scale 2, impairments of physical/mental functioning; Scale 3, impairments of social functioning; Scale 4, impairments of working ability). A total score is calculated for each of the individual scales. Item (6) “Who primarily took care of your child during the night?” is not assigned to any of the five components and delivers relevant clinical anamnestic information. Normative values are available for the HOST scales, based on data from a sample of 214 severely affected parents, with higher scores indicating worse symptoms and consequences of sleep disturbances.

The assessment was provided to parents/caregivers of individuals with AS only and takes approximately 15 minutes to complete. Parents/caregivers could choose to complete the assessment at home after demonstration in the clinic. When it was decided that the assessment would be completed at home, it was required to be finalized up to 4 weeks after the first clinic visit and within the 4 weeks before the second clinic visit.

### CSDI

The Composite Sleep Disturbance Index (CSDI) is based on allocating scores according to the frequency and severity of sleep problems as reported by parents in the sleep diary. It was first used by Richman and Graham (1971) [14] where items on the Behavior Screening Questionnaire were combined to produce the CSDI to reflect the severity of settling and night waking problems. Since that time, it has been used in a number of studies (especially those concerned with children with intellectual disabilities) [15–18] as a means of diary data reduction for assessing the severity of problems and assessing change in response to treatment. It has also been used as a means of dividing sleeplessness problems into severity bands [19, 20]. Quine *et al.* (1991) reported high internal reliability and showed that the measure was sensitive to change. The precise items included vary according to each study, but all have included measuring settling and night waking frequency and duration. Frequency of early waking and co-sleeping have not always been included.

The six items selected in FREESIAS (settling problems, night waking, early waking [before 5 am], co‑sleeping [for all/most of the night] measured in terms of weekly frequency and settling, and night waking problems in terms of the nightly duration of the problem) have been used in a previous randomized controlled trial of behavioral intervention in children with autism [21] in which the CSDI was calculated using the below scoring:

Score 0: If frequency is <1 per week or duration is a few minutes

Score 1: If frequency is 1–2 times per week or duration is ≤30 minutes

Score 2: If frequency is ≥3 times per week or duration is >31 minutes

With six items and a maximum score of 2 for each item, scores range from 0 to 12.

The questionnaire was provided to the parents/caregivers of participants with AS only. It takes approximately 15 to 20 minutes to complete. Parents/caregivers could choose to complete the assessment at home after demonstration in the clinic. When it was decided that the assessment would be completed at home, it was required to be finalized up to 4 weeks after Clinic Visit 1 and within the 4 weeks before Clinic Visit 2.

### PSQI

The Pittsburgh Sleep Quality Index (PSQI) [22] is a self-rated questionnaire, which assesses sleep quality and disturbances over a 1-month time interval. Nineteen self-rated questions assess a variety of factors relating to sleep quality, including estimates of sleep duration and latency and of the frequency and severity of specific sleep-related problems. The 19 individual items generate seven “component” scores: subjective sleep quality, sleep latency, sleep duration, habitual sleep efficiency, sleep disturbances, use of sleeping medication, and daytime dysfunction, each weighted equally on a scale from 0 to 3. Higher scores indicate worse sleep quality. The sum of scores for these seven components yields one global PSQI score, which has a range of 0 to 21. The seven components of the PSQI are standardized versions of areas routinely assessed in clinical interviews of patients with sleep/wake complaints.

The assessment was provided to parents/caregivers of individuals with AS only and takes approximately 5–10 minutes to complete. Parents/caregivers could choose to complete the assessment at home after demonstration in the clinic. When it was decided that the assessment would be completed at home, it was required to be finalized up to 4 weeks after Clinic Visit 1 and within the 4 weeks before Clinic Visit 2.

### ESS

The Epworth Sleepiness Scale (ESS) [23] is a self-administered questionnaire with eight questions. Respondents are asked to rate their usual chances of dozing off or falling asleep while engaged in eight different activities on a scale from 0 to 3. Most people engage in those activities at least occasionally, although not necessarily every day. The ESS score (the sum of eight item scores) ranges from 0 to 24. Higher ESS scores indicate higher average sleep propensity in daily life or increased “daytime sleepiness”. ESS scores of 11 to 24 represent increasing levels of “excessive daytime sleepiness”. The 1997 version of the ESS is the standard version that can be used by most adults.

The assessment was provided to parents/caregivers of individuals with AS only and takes approximately 5 minutes to complete. Parents/caregivers could choose to complete the assessment at home after demonstration in the clinic. When it was decided that the assessment would be completed at home, it was required to be finalized up to 4 weeks after Clinic Visit 1 and within the 4 weeks before Clinic Visit 2.

### ABC-2-C

The Aberrant Behavior Checklist Second Edition – Community Version (ABC-2-C) is an updated, empirically-derived, validated 58-item caregiver-completed rating scale that measures the severity of a range of maladaptive behaviors commonly observed in children, adolescents, and adults with intellectual and developmental disabilities [24]. It is designed for use in individuals who are not residing in institutional settings. The checklist assesses symptoms across five domains: irritability, social withdrawal, stereotypic behavior, hyperactive/noncompliance, and inappropriate speech. Since most of the participants in this study are nonverbal, the “Inappropriate speech” subscale was not completed. The total raw score for each of the other four domains will be calculated and analyzed.

The assessment was provided to the parents/caregivers of individuals with AS only and takes approximately 10 to 15 minutes to complete. Parents/caregivers could choose to complete the assessment at home after demonstration in the clinic. When it was decided that the assessment would be completed at home, it was required to be finalized up to 4 weeks after Clinic Visit 1 and within the 4 weeks before Clinic Visit 2.

### FMS

The Functional Mobility Scale (FMS) is a questionnaire that allows scoring of functional mobility over three distinct distances, chosen to represent mobility in the home, at school, and in the wider community. The FMS has previously been demonstrated to be both valid and reliable in a consecutive population sample of 310 children with cerebral palsy. The FMS was useful for discriminating between large groups of children with varying levels of disabilities and functional mobility and sensitive to detect change after operative intervention. The scale is designed to classify functional mobility, which considers both self-initiated movement as well as assisted movement and passive mobility in a powered wheelchair. The scale is used to rate walking ability at three specific distances: 5, 50, and 500 meters or yards. The scale is designed to be completed by a physician or therapist but may also be completed by the parent after receiving detailed instructions. The rater is asked to rate the usual walking ability of the child at the three distances defined in relation to the absence or presence of a need for assistive devices, such as walking sticks, or crutches, or mobility aids such as a wheelchair. The questionnaire consists of three items, each with a score of 1 (“uses wheelchair”) to 6 (“independent on all surfaces”). The maximum possible score is 18.

The assessment takes about 5 to 10 minutes to complete. It was used for participants with AS and TDC.

### PedsQL™-FIM

The Pediatric Quality of Life Inventory™ Family Impact Module (PedsQL™-FIM), Version 2 [25, 26] is a 36-item, informant-based measure that will be completed by the parent/caregiver. The instrument was developed to measure parent and family functioning. It encompasses six scales covering physical functioning (6 items), emotional functioning (5 items), social functioning (4 items), cognitive functioning (5 items), communication (3 items), and worry (5 items) and two scales measuring parent-reported family functioning, daily activities (3 items), and family relationships (5 items). The acute form, using a recall period of 7 days, will be employed in this trial.

Each item utilizes a 5-point response scale, ranging from “never (a problem)” (0) to “almost always (a problem)” (4). Items are then reverse-scored and linearly transformed to a 0–100 scale (0 = 100, 1 = 75, 2 = 50, 3 = 25, 4 = 0), such that higher scores indicate better functioning (less negative impact).

In addition to the eight scale scores, a total score, a parent/caregiver health-related quality of life (HRQoL) summary score and a family summary score can also be computed by averaging across the relevant domains.

The assessment was provided to parents/caregivers of individuals with AS only and takes approximately 15 to 20 minutes to complete. Parents/caregivers could choose to complete the assessment at home after demonstration in the clinic for the time points indicated in the Schedule of Activities. When it was decided that the assessment would be completed at home, it was required to be finalized up to 4 weeks after Clinic Visit 1 and within the 4 weeks before Clinic Visit 2.

### PedsQL™ 4.0 Core

The Pediatric Quality of Life Inventory™ Generic Core Scales, Version 4.0 (PedsQL™ 4.0 Core) is a self-reported assessment encompassing four core scale domains: Physical Functioning*,* Emotional Functioning*,* Social Functioning*,* and School Functioning [26]*.*

The acute form for toddlers aged 2–4 years, using a recall period of 7 days, was employed. Different age-appropriate versions were utilized depending on the age of the individual. The proxy report versions to be completed by the parent/caregiver were used.

Each item utilizes a 5-point response scale ranging from “never (a problem)” (0) to “almost always (a problem)” (4). Items are then reverse-scored and linearly transformed to a 0−100 scale (0 = 100, 1 = 75, 2 = 50, 3 = 25, 4 = 0), such that higher scores indicate better HRQoL.

In addition to the four scale scores (Physical Functioning, Emotional Functioning, Social Functioning, School/Work Functioning), a Psychosocial Health Summary score, a Physical Health Summary score, and a total score can also be computed by averaging across the relevant domains.

The assessment was provided to the parents/caregivers of participants with AS only. It takes approximately 15 to 20 minutes to complete. Parents/caregivers could choose to complete the assessment at home after demonstration in the clinic. When it was decided that the assessment would be completed at home, it was required to be finalized up to 4 weeks after Clinic Visit 1 and within the 4 weeks before Clinic Visit 2.

### EQ-5D-5L

The European Quality of Life 5-Dimensions Questionnaire-Five Levels (EQ-5D-5L) is a validated, self‑reported health status questionnaire that is used to calculate a health status utility score for use in health economic analyses [27–30]. There are two components to the EQ-5D-5L: a 5-item health state profile that assesses mobility, self‑care, usual activities, pain/discomfort, and anxiety/depression, as well as a visual analogue scale (VAS) that measures health state. Published weighting systems allow for creation of a single composite score of an individual’s health status. Overall, scores range from 0 to 1, with low scores representing a higher level of dysfunction.

Parents/caregivers completed the measure (reporting on their own health status) to generate health status data to support pharmacoeconomic evaluations.

The assessment was provided to the parents/caregivers of individuals with AS only and takes approximately 2 minutes to complete. Parents/caregivers could choose to complete the assessment at home after demonstration in the clinic. When it was decided that the assessment would be completed at home, it was required to be finalized up to 4 weeks after Clinic Visit 1 and within the 4 weeks before Clinic Visit 2.

### EQ-5D-Y

The European Quality of Life 5-Dimensions Questionnaire-Youth (EQ-5D-Y) Proxy is a validated, self‑reported health status questionnaire that is used to calculate a health status utility score for use in health economic analyses. The EQ-5D-Y is based on the EQ-5D-Three Levels and consists of two pages: the EQ-5D descriptive system and the EQ VAS.

The Proxy Version 1 of EQ-5D-Y was used in this study: the caregiver (the proxy) is asked to rate the child’s/adolescent’s HRQoL in their (the proxy’s) opinion.

The EQ-5D-Y descriptive system comprises the following five dimensions: mobility, looking after oneself, doing usual activities, having pain or discomfort, and feeling worried, sad, or unhappy. Each dimension has three levels: no problems, some problems, and a lot of problems. The caregiver is asked to indicate their child’s state by ticking the box next to the most appropriate statement in each of the five dimensions. This decision results in a one-digit number that expresses the level selected for that dimension. The digits for the five dimensions can be combined into a five-digit number that describes the younger patient’s health state.

The EQ VAS records the caregiver’s assessment of the patient’s health on a vertical VAS where the endpoints are labelled “The best health you can imagine” and “The worst health you can imagine”. Overall, scores range from 0 to 1, with low scores representing a higher level of dysfunction.

The assessment will be provided to the parents/caregivers of AS patients only and will take approximately 5 minutes to complete. Parents/caregivers may choose to complete the assessment at home after demonstration in the clinic. When it was decided that the assessment would be completed at home, it was required to be finalized up to 4 weeks after Clinic Visit 1 and within the 4 weeks before Clinic Visit 2.

## Digital health technologies (DHTs)

### Seizure diary

Seizure diaries have been found to be useful for patient-based prediction of subsequent seizures [31, 32].

A seizure diary was implemented on a smartphone device and handed out to caregivers to collect information on seizure type, frequency (date/time of occurrence), duration, and recovery time, based on guidance from the National Institutes of Neurological Diseases and Stroke (NINDS)[33] on common data element forms to encourage consistent data collection in epilepsy clinical trials.

The seizure diary was provided only to parents/caregivers of participants with AS for completion at home after initial training at the clinic. The questions were shown on the smartphone once triggered by the parents following an observed seizure event. The completion of an entry took about 2 minutes.

The seizure types were recorded based on operational classification guidelines of the International League Against Epilepsy (ILAE) [34], and mapped to a layman’s term in the caregiver’s own language that was agreed upon with the Investigators and displayed in the app.

**Screenshots of the seizure diary questions**


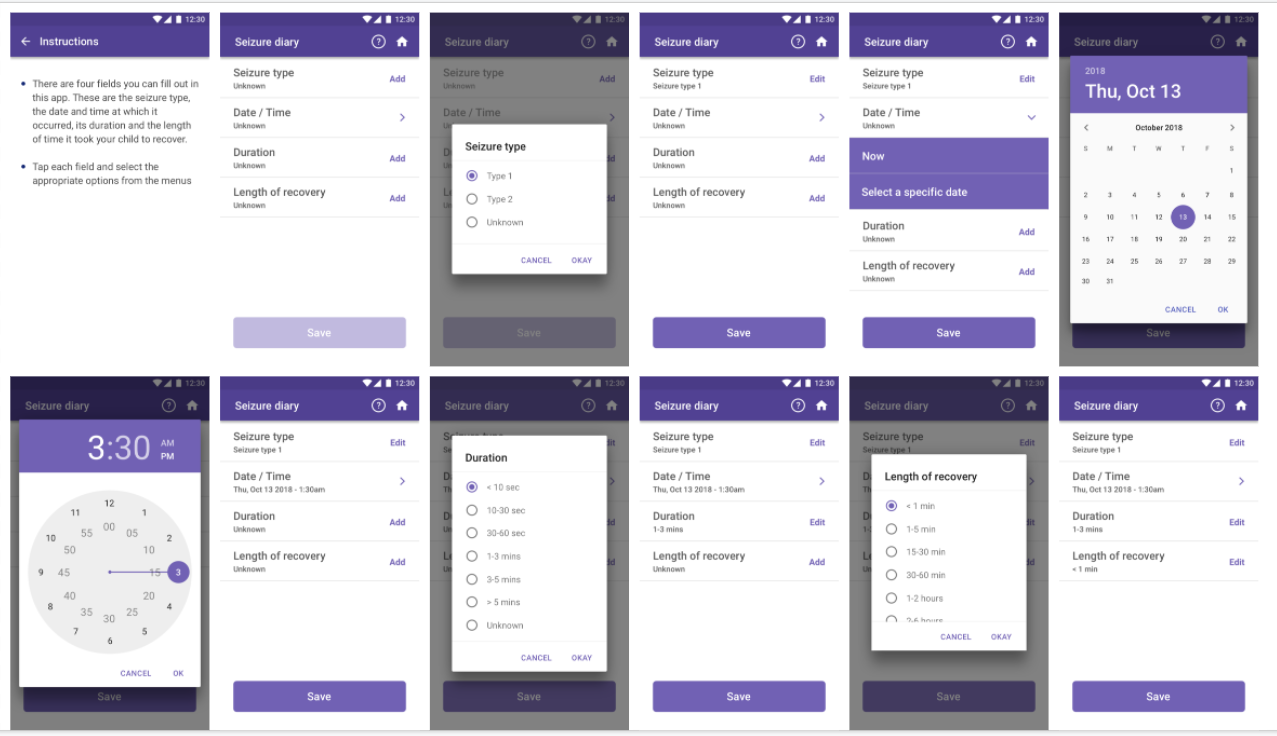


### Sleep diary

Caregivers of individuals with AS and TDC were asked for daily completion of a sleep diary with questions concerning the nighttime sleep of the participants, which took approximately 5 minutes to complete. Questions related to sleep quality of the participant and caregiver, time in bed, time to fall asleep, number and duration of nocturnal awakenings, and when the participant woke up. Caregivers were asked to estimate the clock time and the duration for any nocturnal awakenings. Caregivers were also allowed to select “unknown” in the number of awakenings, in which case they were asked to estimate the number of awakenings and the longest uninterrupted sleep period. However, entering an estimate was not mandatory and thus resulted in missing values if not provided. The questions were scheduled automatically by the smartphone app for completion at home after initial training in the clinic.

**Screenshots of the sleep diary questions**

**
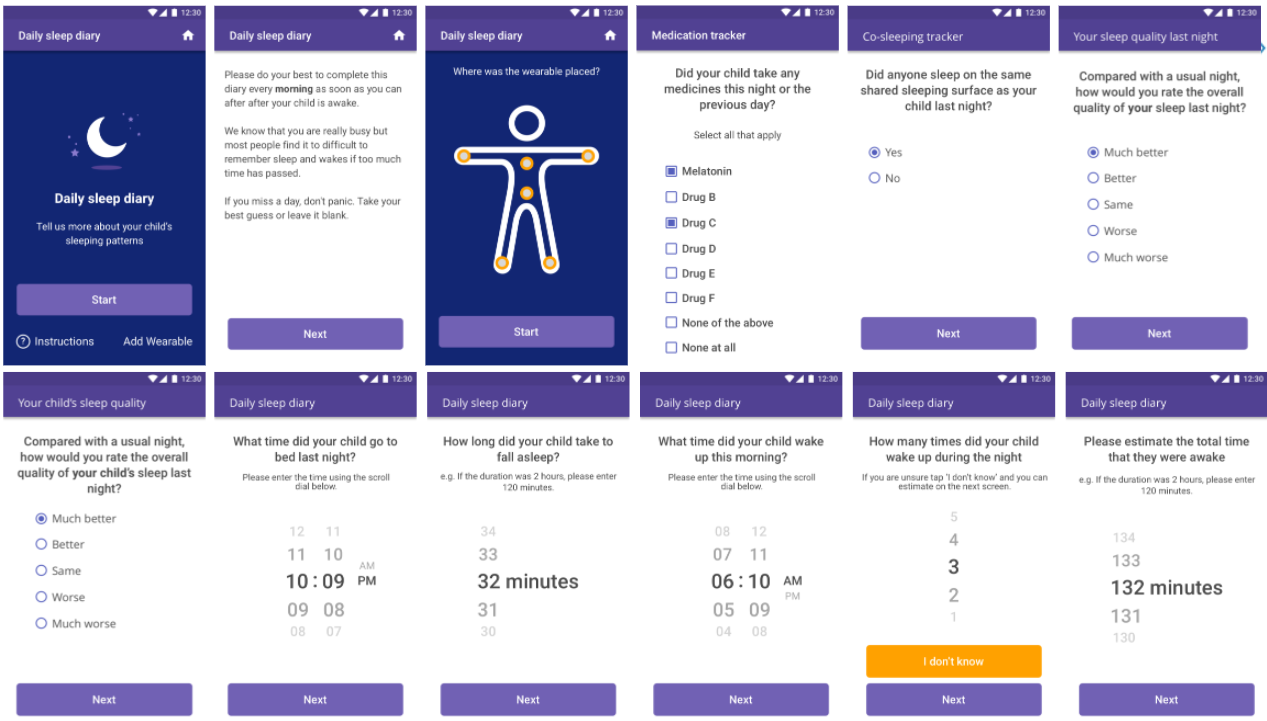
**

### Sleep mat

Parents/caregivers of individuals with AS and TDC were provided with an Emfit sleep mat [35]. The sleep mat is a highly sensitive technology for recording vibrations (a ballistiocardiogram), which can be caused by body movements or physiologic processes including heartbeat and respiration. These data are used to estimate bed presence and sleep/wake state.

Parents/caregivers were asked to place the sleep mat under the mattress of the participant’s bed and leave the hardware plugged in to a mains electrical socket from the screening visit until the end of the study. When plugged in, the sleep mat continuously monitors bed presence and, when an individual is detected in the bed, it initiates a ballistiocardiogram recording that concludes when a bed exit is detected. The hardware is connected to a cellular-data network for automatic data upload to a secure cloud-server.

### Actigraph

Parents/caregivers of individuals with AS and TDC were provided with an ActiGraph GT9X Link [36]. The ActiGraph GT9X Link is a watch that records three-axis accelerometer, gyroscope, and magnetometer for estimating movement, rotation, and body position. It can be worn on the wrist or ankle, or the watch face can be detached so it can be placed in a pocket. It includes a docking station and hub with a connection to a cellular-data network for data upload to a secure cloud-server.

In discussions with participants prior to the study, feedback was received that some participants may not tolerate wearing a watch on the skin and were unlikely to tolerate a wearable for long periods of time. For these reasons parents/caregivers were permitted to choose where to place the watch face for the recording (left/right wrist or ankle, or in a pocket), limited the duration of the recording to a monitoring window from 10 days before until 1 day after each home visit, and asked that the participant only wear the actigraph from 30 minutes prior to going to bed up until they leave the bed the next morning. The parent/caregiver was instructed to place the actigraph on the docking station the morning after a recording. The hub then automatically uploaded the data to a secure cloud-server.

### Overnight EEG and limited polysomnography (PSG)

Overnight EEG/limited PSG recordings were performed in the participants’ home. Per recording, an EEG technologist visited the home in the afternoon, connected the EEG electrodes and other sensors, ensured good quality recording, instructed caregivers about the overnight assessment, and then left the home to stay in a hotel nearby to be able to return in case of emerging technical problems (e.g., loss of electrode connection to scalp). Participants slept in their beds while wearing the limited PSG equipment. Recording continued through the night, and into the following day’s morning waking hours. The EEG technologist returned to the home, and the equipment was removed after the morning waking recording period on the second day of recording. With each recording, the EEG technologist marked 10 minutes of awake EEG data in the afternoon of the first day and the morning of the second day for subsequent quantitative analyses.

Electrophysiologic data were recorded with TrackItTm Mk3 (Lifelines Neuro, sampling rate 400 Hz) with the following sensors: 19 EEG channels (10–20 montage, FC5 system reference) [37], one electrocardiogram channel referenced to the EEG reference, two electrooculogram channels (left under eye and right above eye), two leg electromyography (EMG; left leg, right leg), one abdominal belt, one chin EMG, one pulse oximeter was discontinued after initial 22 recordings. This setup was referred to as “limited PSG” to acknowledge that not all sensors of a PSG were deployed. Participants were monitored with an infrared camera during sleep with continuous video monitoring

For this publication, 10 minutes of awake EEG data recorded on the first day of Home Visit 1 were analyzed quantitatively following the procedures described previously to extract EEG delta-band power [38]. Analyses included artifact rejection (semi-manual including rejection of sections with artifacts and rejection of artifactual independent components) and spectral analysis to extract power in the delta frequency range.

## Mapping of COAs and DHTs to AS symptomatic domains

|  | Key AS domains measured | | | | | | | | |  | |
| --- | --- | --- | --- | --- | --- | --- | --- | --- | --- | --- | --- |
|  | **Seizures** | **Sleep** | **Motor function** | **Communication** | **Cognition** | **Self-care** | **Maladaptive behaviors** | **Quality of life** | **Overall AS** | **Participant focus** | **Caregiver focus** |
| Bayley-III |  |  | X | X | X |  |  |  |  | X |  |
| Vineland-3 |  |  | X | X |  | X | X |  |  | X |  |
| CGI-S |  |  |  |  |  |  |  |  | X | X |  |
| Communication Matrix |  |  |  | X |  |  |  |  |  | X |  |
| SNAKE |  | X |  |  |  |  |  |  |  | X |  |
| HOST |  | X |  |  |  |  |  |  |  |  | X |
| CSDI |  | X |  |  |  |  |  |  |  | X |  |
| PSQI |  | X |  |  |  |  |  |  |  |  | X |
| ESS |  | X |  |  |  |  |  |  |  |  | X |
| ABC-2-C |  |  |  |  |  |  | X |  |  | X |  |
| FMS |  |  | X |  |  |  |  |  |  | X |  |
| Seizure diary | X |  |  |  |  |  |  |  |  | X |  |
| Sleep diary |  | X |  |  |  |  |  |  |  | X | X |
| Sleep mat |  | X |  |  |  |  |  |  |  | X |  |
| Actigraph |  | X |  |  |  |  |  |  |  | X |  |
| Overnight EEG | X | X |  |  |  |  |  |  |  | X |  |
| PedsQL™-FIM |  |  |  |  |  |  |  | X |  |  | X |
| PedsQL™ 4.0 Core |  |  |  |  |  |  |  | X |  | X |  |
| EQ-5D-5L |  |  |  |  |  |  |  | X |  |  | X |
| EQ-5D-Y |  |  |  |  |  |  |  | X |  | X |  |
| *ABC-2-C* Aberrant Behavior Checklist Second Edition – Community Version, *AS* Angelman syndrome, *Bayley-III* Bayley Scales of Infant and Toddler Development® – Third Edition, *CGI-S* Clinical Global Impression – Severity, *CSDI* Composite Sleep Disturbance Index, *EEG* electroencephalogram, *EQ-5D-5L* European Quality of Life 5-Dimensions Questionnaire-Five Levels, *EQ-5D-Y* European Quality of Life 5-Dimensions Questionnaire-Youth, *ESS* Epworth Sleepiness Scale, *FMS* Functional Mobility Scale, *HOST* holistic assessment of sleep and daily troubles in parents of children with severe psychomotor impairment, *PedsQL™ 4.0 Core* Pediatric Quality of Life Inventory™ Generic Core Scales, Version 4.0, *PedsQL™-FIM* Pediatric Quality of Life Inventory™ Family Impact Module, *PSQI* Pittsburgh Sleep Quality Index, *SNAKE* Schlaffragebogen für Kinder mit Neurologischen und Anderen Komplexen Erkrankungen (Sleep Questionnaire for Children with Severe Psychomotor Impairment), *Vineland-3* Vineland Adaptive Behavior Scales® – Third Edition | | | | | | | | | | | |

# Supplementary references

1. Bayley N. Manual for the Bayley Scales of Infant Development. New York: Psychological Corporation; 1969.

2. Sadhwani A, Wheeler A, Gwaltney A, Peters SU, Barbieri-Welge RL, Horowitz LT, et al. Developmental skills of individuals with Angelman syndrome assessed using the Bayley-III. J Autism Dev Disord. 2022; doi: 10.1007/s10803-020-04861-1.

3. Albers CA, Grieve AJ. Test review: Bayley, N.(2006). Bayley Scales of Infant and Toddler Development – Third Edition. San Antonio, TX: Harcourt assessment. J Psychoeduc Assess. 2007;25:180–190; doi: 10.1177/0734282906297199.

4. Gentile JK, Tan W-H, Horowitz LT, Bacino CA, Skinner SA, Barbieri-Welge RL, et al. A neurodevelopmental survey of Angelman syndrome with genotype-phenotype correlations. J Dev Behav Pediatr. 2010;31:592–601.

5. Sparrow SS, Cicchetti DV, Saulnier CA. Vineland Adaptive Behavior Scales, Third Edition (Vineland-3). 2016. <https://www.pearsonassessments.com/store/usassessments/en/Store/Professional-Assessments/Behavior/Adaptive/Vineland-Adaptive-Behavior-Scales-%7C-Third-Edition/p/100001622.html>. Accessed 12 May 2022.

6. Rowland C. Communication Matrix especially for parents. 2004. <https://documents.nationaldb.org/products/Parent-Comm-Matrix-Final.pdf>. Accessed 12 May 2022.

7. Rowland C. Using the Communication Matrix to assess expressive skills in early communicators. Commun Disord Q. 2011;32:190–201.

8. Rowland C. Communication Matrix: Description, research basis and data. 2012. <https://communicationmatrix.org/Uploads/Pdfs/CommunicationMatrixDataandResearchBasis.pdf>. Accessed 12 May 2022.

9. Rowland C, Fried-Oken M. Communication Matrix: a clinical and research assessment tool targeting children with severe communication disorders. J Pediatr Rehabil Med. 2010;3:319–29.

10. Communicationmatrix.org. Welcome to the Communication Matrix. <https://www.communicationmatrix.org/>. Accessed 12 May 2022.

11. Quinn ED, Rowland C. Exploring expressive communication skills in a cross-sectional sample of children and young adults with Angelman syndrome. Am J Speech Lang Pathol. 2017;26:369–82.

12. Blankenburg M, Tietze A-L, Hechler T, Hirschfeld G, Michel E, Koh M, et al. Snake: the development and validation of a questionnaire on sleep disturbances in children with severe psychomotor impairment. Sleep Med 2013;14:339–351.

13. Tietze AL, Zernikow B, Otto M, Hirschfeld G, Michel E, Koh M, et al. The development and psychometric assessment of a questionnaire to assess sleep and daily troubles in parents of children and young adults with severe psychomotor impairment. Sleep Med. 2014;15:219–27.

14. Richman N, Graham PJ. A behavioural screening questionnaire for use with three-year-old children. Preliminary findings. J Child Psychol Psychiatry. 1971;12:5–33.

15. Quine LYN, Pahl JAN. Examining the causes of stress in families with severely mentally handicapped children. Br J Soc Work. 1985;15:501–17.

16. Quine L. Sleep problems in children with mental handicap. J Ment Defic Res. 1991;35 (Pt 4):269–90.

17. Wiggs L, Stores G. Factors affecting parental reports of the sleep patterns of children with severe learning disabilities. Br J Health Psychol. 1998;3:345–59.

18. Montgomery P, Stores G, Wiggs L. The relative efficacy of two brief treatments for sleep problems in young learning disabled (mentally retarded) children: a randomised controlled trial. Arch Dis Child. 2004;89:125–30.

19. Wiggs L, Stores G. Severe sleep disturbance and daytime challenging behaviour in children with severe learning disabilities. J Intellect Disabil Res. 1996;40:518–28.

20. Brylewski J, Wiggs L. Sleep problems and daytime challenging behaviour in a community-based sample of adults with intellectual disability. J Intellect Disabil Res. 1999;43:504–12.

21. Gringras P, Nir T, Breddy J, Frydman-Marom A, Findling RL. Efficacy and safety of pediatric prolonged-release melatonin for insomnia in children with autism spectrum disorder. J Am Acad Child Adolesc Psychiatry. 2017;56:948–57.e4.

22. Buysse DJ, Reynolds III CF, Monk TH, Berman SR, Kupfer DJ. The Pittsburgh Sleep Quality Index: a new instrument for psychiatric practice and research. Psychiatry Res. 1989;28:193–213.

23. Johns M, Hocking BM. Daytime sleepiness and sleep habits of Australian workers. Sleep. 1997;20:844–9.

24. Aman MG, Kasper W, Manos G, Mathew S, Marcus R, Owen R, et al. Line-item analysis of the Aberrant Behavior Checklist: results from two studies of aripiprazole in the treatment of irritability associated with autistic disorder. J Child Adolesc Psychopharmacol. 2010;20:415–22.

25. Varni JW, Sherman SA, Burwinkle TM, Dickinson PE, Dixon P. The PedsQL™ family impact module: preliminary reliability and validity. Health Qual Life Outcomes. 2004;2:1–6.

26. Varni JW, Limbers CA, Burwinkle TM. Parent proxy-report of their children's health-related quality of life: an analysis of 13,878 parents' reliability and validity across age subgroups using the PedsQL 4.0 Generic Core Scales. Health Qual Life Outcomes. 2007;5:2.

27. EuroQol Group. EuroQol--a new facility for the measurement of health-related quality of life. Health Policy. 1990;16:199–208.

28. Brooks R. EuroQol: the current state of play. Health Policy. 1996;37:53–72.

29. Herdman M, Gudex C, Lloyd A, Janssen MF, Kind P, Parkin D, et al. Development and preliminary testing of the new five-level version of EQ-5D (EQ-5D-5L). Qual Life Res. 2011;20:1727–36.

30. Janssen MF, Pickard AS, Golicki D, Gudex C, Niewada M, Scalone L, et al. Measurement properties of the EQ-5D-5L compared to the EQ-5D-3L across eight patient groups: a multi-country study. Qual Life Res. 2013;22:1717–27.

31. Fisher RS, Blum DE, DiVentura B, Vannest J, Hixson JD, Moss R, et al. Seizure diaries for clinical research and practice: limitations and future prospects. Epilepsy Behav. 2012;24:304–10.

32. Hall CB, Lipton RB, Tennen H, Haut SR. Early follow-up data from seizure diaries can be used to predict subsequent seizures in same cohort by borrowing strength across participants. Epilepsy Behav. 2009;14:472–5.

33. National Institutes of Neurological Diseases and Stroke. Data Standards. <https://www.commondataelements.ninds.nih.gov/epilepsy#pane-145>. Accessed 12 May 2022.

34. Fisher RS, Cross JH, French JA, Higurashi N, Hirsch E, Jansen FE, et al. Operational classification of seizure types by the International League Against Epilepsy: Position Paper of the ILAE Commission for Classification and Terminology. Epilepsia. 2017;58:522–30.

35. Emfit.com. Contact-Free Sleep Tracking and Analyzing.
<https://emfit.com/>. Accessed Dec 2022.

36. actigraphcorp. ActiGraph is a leading provider of medical-grade wearable technology solutions for the global scientific community.
<https://actigraphcorp.com/>. Accessed 12 May 2022.

37. Oostenveld R, Praamstra P. The five percent electrode system for high-resolution EEG and ERP measurements. Clin Neurophysiol. 2001;112:713–719.

38. Frohlich J, Reiter LT, Saravanapandian V, DiStefano C, Huberty S, Hyde C, et al. Mechanisms underlying the EEG biomarker in Dup15q syndrome. Mol Autism. 2019;10:29.

# Supplementary tables and figures

**Table S1** Previous and concurrent medical history (≥5% in either AS or TDC group)

|  | **All AS (*N* = 55) (%)** | **All TDC (*N* = 20) (%)** | **AS 1–4 years (*N* = 16) (%)** | **AS 5–12 years (*N* = 27) (%)** | **AS 18+ years (*N* = 12) (%)** | **TDC 1–4 years**  **(*N* = 9) (%)** | **TDC 5–12 years (*N* = 11) (%)** | **AS deletion (*N* = 40) (%)** | **AS nondeletion (*N* = 15) (%)** |
| --- | --- | --- | --- | --- | --- | --- | --- | --- | --- |
| Patent ductus arteriosus | 2 (3.6) | 1 (5.0) | 0 (0.0) | 1 (3.7) | 1 (8.3) | 0 (0.0) | 1 (9.1) | 2 (5.0) | 0 (0.0) |
| Cryptorchism | 0 (0.0) | 2 (10.0) | 0 (0.0) | 0 (0.0) | 0 (0.0) | 0 (0.0) | 2 (18.2) | 0 (0.0) | 0 (0.0) |
| Ankyloglossia congenital | 0 (0.0) | 1 (5.0) | 0 (0.0) | 0 (0.0) | 0 (0.0) | 1 (11.1) | 0 (0.0) | 0 (0.0) | 0 (0.0) |
| Strabismus | 22 (40.0) | 1 (5.0) | 5 (31.3) | 13 (48.1) | 4 (33.3) | 0 (0.0) | 1 (9.1) | 20 (50.0) | 2 (13.3) |
| Astigmatism | 4 (7.3) | 1 (5.0) | 2 (12.5) | 2 (7.4) | 0 (0.0) | 0 (0.0) | 1 (9.1) | 3 (7.5) | 1 (6.7) |
| Hypermetropia | 1 (1.8) | 2 (10.0) | 0 (0.0) | 1 (3.7) | 0 (0.0) | 0 (0.0) | 2 (18.2) | 1 (2.5) | 0 (0.0) |
| Myopia | 1 (1.8) | 1 (5.0) | 1 (6.3) | 0 (0.0) | 0 (0.0) | 0 (0.0) | 1 (9.1) | 1 (2.5) | 0 (0.0) |
| Gastrooesophageal reflux disease | 33 (60.0) | 1 (5.0) | 10 (62.5) | 17 (63.0) | 6 (50.0) | 0 (0.0) | 1 (9.1) | 22 (55.0) | 11 (73.3) |
| Constipation | 30 (54.5) | 2 (10.0) | 10 (62.5) | 11 (40.7) | 9 (75.0) | 2 (22.2) | 0 (0.0) | 23 (57.5) | 7 (46.7) |
| Dysphagia | 5 (9.1) | 0 (0.0) | 2 (12.5) | 2 (7.4) | 1 (8.3) | 0 (0.0) | 0 (0.0) | 4 (10.0) | 1 (6.7) |
| Inguinal hernia | 5 (9.1) | 0 (0.0) | 2 (12.5) | 2 (7.4) | 1 (8.3) | 0 (0.0) | 0 (0.0) | 3 (7.5) | 2 (13.3) |
| Vomiting | 4 (7.3) | 0 (0.0) | 2 (12.5) | 2 (7.4) | 0 (0.0) | 0 (0.0) | 0 (0.0) | 3 (7.5) | 1 (6.7) |
| Umbilical hernia | 3 (5.5) | 0 (0.0) | 0 (0.0) | 2 (7.4) | 1 (8.3) | 0 (0.0) | 0 (0.0) | 1 (2.5) | 2 (13.3) |
| Hernia | 1 (1.8) | 1 (5.0) | 0 (0.0) | 1 (3.7) | 0 (0.0) | 0 (0.0) | 1 (9.1) | 1 (2.5) | 0 (0.0) |
| Drug hypersensitivity | 6 (10.9) | 0 (0.0) | 1 (6.3) | 1 (3.7) | 4 (33.3) | 0 (0.0) | 0 (0.0) | 5 (12.5) | 1 (6.7) |
| Food allergy | 4 (7.3) | 0 (0.0) | 1 (6.3) | 2 (7.4) | 1 (8.3) | 0 (0.0) | 0 (0.0) | 4 (10.0) | 0 (0.0) |
| Milk allergy | 4 (7.3) | 0 (0.0) | 1 (6.3) | 3 (11.1) | 0 (0.0) | 0 (0.0) | 0 (0.0) | 4 (10.0) | 0 (0.0) |
| Seasonal allergy | 4 (7.3) | 0 (0.0) | 3 (18.8) | 0 (0.0) | 1 (8.3) | 0 (0.0) | 0 (0.0) | 3 (7.5) | 1 (6.7) |
| Hypersensitivity | 3 (5.5) | 0 (0.0) | 2 (12.5) | 0 (0.0) | 1 (8.3) | 0 (0.0) | 0 (0.0) | 3 (7.5) | 0 (0.0) |
| Allergy to animal | 2 (3.6) | 1 (5.0) | 0 (0.0) | 2 (7.4) | 0 (0.0) | 1 (11.1) | 0 (0.0) | 1 (2.5) | 1 (6.7) |
| Pneumonia | 9 (16.4) | 0 (0.0) | 2 (12.5) | 3 (11.1) | 4 (33.3) | 0 (0.0) | 0 (0.0) | 7 (17.5) | 2 (13.3) |
| Ear infection | 7 (12.7) | 2 (10.0) | 2 (12.5) | 4 (14.8) | 1 (8.3) | 2 (22.2) | 0 (0.0) | 6 (15.0) | 1 (6.7) |
| Otitis media | 7 (12.7) | 1 (5.0) | 1 (6.3) | 3 (11.1) | 3 (25.0) | 1 (11.1) | 0 (0.0) | 6 (15.0) | 1 (6.7) |
| Pharyngitis streptococcal | 4 (7.3) | 2 (10.0) | 1 (6.3) | 3 (11.1) | 0 (0.0) | 1 (11.1) | 1 (9.1) | 2 (5.0) | 2 (13.3) |
| Urinary tract infection | 3 (5.5) | 1 (5.0) | 0 (0.0) | 2 (7.4) | 1 (8.3) | 0 (0.0) | 1 (9.1) | 2 (5.0) | 1 (6.7) |
| Otitis media acute | 1 (1.8) | 1 (5.0) | 1 (6.3) | 0 (0.0) | 0 (0.0) | 0 (0.0) | 1 (9.1) | 1 (2.5) | 0 (0.0) |
| Pharyngitis | 1 (1.8) | 1 (5.0) | 0 (0.0) | 0 (0.0) | 1 (8.3) | 1 (11.1) | 0 (0.0) | 1 (2.5) | 0 (0.0) |
| Influenza | 0 (0.0) | 1 (5.0) | 0 (0.0) | 0 (0.0) | 0 (0.0) | 0 (0.0) | 1 (9.1) | 0 (0.0) | 0 (0.0) |
| Fall | 2 (3.6) | 1 (5.0) | 0 (0.0) | 1 (3.7) | 1 (8.3) | 1 (11.1) | 0 (0.0) | 1 (2.5) | 1 (6.7) |
| Contusion | 1 (1.8) | 1 (5.0) | 0 (0.0) | 1 (3.7) | 0 (0.0) | 1 (11.1) | 0 (0.0) | 0 (0.0) | 1 (6.7) |
| Feeding disorder | 5 (9.1) | 0 (0.0) | 1 (6.3) | 4 (14.8) | 0 (0.0) | 0 (0.0) | 0 (0.0) | 4 (10.0) | 1 (6.7) |
| Poor feeding infant | 3 (5.5) | 0 (0.0) | 0 (0.0) | 2 (7.4) | 1 (8.3) | 0 (0.0) | 0 (0.0) | 2 (5.0) | 1 (6.7) |
| Weight gain poor | 3 (5.5) | 0 (0.0) | 1 (6.3) | 2 (7.4) | 0 (0.0) | 0 (0.0) | 0 (0.0) | 3 (7.5) | 0 (0.0) |
| Foot deformity | 9 (16.4) | 0 (0.0) | 1 (6.3) | 8 (29.6) | 0 (0.0) | 0 (0.0) | 0 (0.0) | 5 (12.5) | 4 (26.7) |
| Scoliosis | 6 (10.9) | 0 (0.0) | 1 (6.3) | 1 (3.7) | 4 (33.3) | 0 (0.0) | 0 (0.0) | 5 (12.5) | 1 (6.7) |
| Toe walking | 2 (3.6) | 1 (5.0) | 0 (0.0) | 2 (7.4) | 0 (0.0) | 0 (0.0) | 1 (9.1) | 1 (2.5) | 1 (6.7) |
| Muscle tightness | 0 (0.0) | 1 (5.0) | 0 (0.0) | 0 (0.0) | 0 (0.0) | 0 (0.0) | 1 (9.1) | 0 (0.0) | 0 (0.0) |
| Tremor | 13 (23.6) | 0 (0.0) | 3 (18.8) | 8 (29.6) | 2 (16.7) | 0 (0.0) | 0 (0.0) | 6 (15.0) | 7 (46.7) |
| Hypotonia | 8 (14.5) | 0 (0.0) | 2 (12.5) | 4 (14.8) | 2 (16.7) | 0 (0.0) | 0 (0.0) | 6 (15.0) | 2 (13.3) |
| Resting tremor | 5 (9.1) | 0 (0.0) | 3 (18.8) | 1 (3.7) | 1 (8.3) | 0 (0.0) | 0 (0.0) | 4 (10.0) | 1 (6.7) |
| Dyskinesia | 4 (7.3) | 0 (0.0) | 2 (12.5) | 2 (7.4) | 0 (0.0) | 0 (0.0) | 0 (0.0) | 3 (7.5) | 1 (6.7) |
| Psychomotor hyperactivity | 4 (7.3) | 0 (0.0) | 1 (6.3) | 2 (7.4) | 1 (8.3) | 0 (0.0) | 0 (0.0) | 2 (5.0) | 2 (13.3) |
| Ataxia | 3 (5.5) | 0 (0.0) | 0 (0.0) | 3 (11.1) | 0 (0.0) | 0 (0.0) | 0 (0.0) | 1 (2.5) | 2 (13.3) |
| Drooling | 3 (5.5) | 0 (0.0) | 1 (6.3) | 1 (3.7) | 1 (8.3) | 0 (0.0) | 0 (0.0) | 3 (7.5) | 0 (0.0) |
| Poor sucking reflex | 3 (5.5) | 0 (0.0) | 2 (12.5) | 1 (3.7) | 0 (0.0) | 0 (0.0) | 0 (0.0) | 2 (5.0) | 1 (6.7) |
| Jaundice neonatal | 3 (5.5) | 0 (0.0) | 1 (6.3) | 0 (0.0) | 2 (16.7) | 0 (0.0) | 0 (0.0) | 0 (0.0) | 3 (20.0) |
| Insomnia | 15 (27.3) | 4 (20.0) | 2 (12.5) | 9 (33.3) | 4 (33.3) | 3 (33.3) | 1 (9.1) | 11 (27.5) | 4 (26.7) |
| Sleep disorder | 17 (30.9) | 0 (0.0) | 6 (37.5) | 11 (40.7) | 0 (0.0) | 0 (0.0) | 0 (0.0) | 12 (30.0) | 5 (33.3) |
| Anxiety | 7 (12.7) | 0 (0.0) | 1 (6.3) | 3 (11.1) | 3 (25.0) | 0 (0.0) | 0 (0.0) | 4 (10.0) | 3 (20.0) |
| Autism spectrum disorder | 3 (5.5) | 0 (0.0) | 0 (0.0) | 3 (11.1) | 0 (0.0) | 0 (0.0) | 0 (0.0) | 0 (0.0) | 3 (20.0) |
| Disruptive mood dysregulation disorder | 3 (5.5) | 0 (0.0) | 1 (6.3) | 2 (7.4) | 0 (0.0) | 0 (0.0) | 0 (0.0) | 2 (5.0) | 1 (6.7) |
| Asthma | 4 (7.3) | 1 (5.0) | 0 (0.0) | 3 (11.1) | 1 (8.3) | 1 (11.1) | 0 (0.0) | 2 (5.0) | 2 (13.3) |
| Sleep apnoea syndrome | 4 (7.3) | 1 (5.0) | 2 (12.5) | 2 (7.4) | 0 (0.0) | 0 (0.0) | 1 (9.1) | 2 (5.0) | 2 (13.3) |
| Bronchial hyperreactivity | 1 (1.8) | 1 (5.0) | 0 (0.0) | 1 (3.7) | 0 (0.0) | 1 (11.1) | 0 (0.0) | 1 (2.5) | 0 (0.0) |
| Rhinitis allergic | 1 (1.8) | 1 (5.0) | 0 (0.0) | 0 (0.0) | 1 (8.3) | 1 (11.1) | 0 (0.0) | 0 (0.0) | 1 (6.7) |
| Eczema | 8 (14.5) | 2 (10.0) | 4 (25.0) | 3 (11.1) | 1 (8.3) | 2 (22.2) | 0 (0.0) | 5 (12.5) | 3 (20.0) |
| Drug eruption | 2 (3.6) | 1 (5.0) | 0 (0.0) | 2 (7.4) | 0 (0.0) | 1 (11.1) | 0 (0.0) | 1 (2.5) | 1 (6.7) |
| Macule | 1 (1.8) | 1 (5.0) | 0 (0.0) | 1 (3.7) | 0 (0.0) | 0 (0.0) | 1 (9.1) | 0 (0.0) | 1 (6.7) |
| Seborrhoeic dermatitis | 1 (1.8) | 1 (5.0) | 0 (0.0) | 0 (0.0) | 1 (8.3) | 1 (11.1) | 0 (0.0) | 1 (2.5) | 0 (0.0) |
| *AS* Angelman syndrome, *TDC* typically developing children | | | | | | | | | |

**Table S2** Seizure history

|  | **All AS**  **(*N* = 55) (%)** | **AS 1–4 years (*N* = 16) (%)** | **AS 5–12 years (*N* = 27) (%)** | **AS 18+ years (*N* = 12) (%)** | **AS deletion  (*N* = 40) (%)** | **AS nondeletion (*N* = 15) (%)** |
| --- | --- | --- | --- | --- | --- | --- |
| **Does patient have epilepsy?** | | | | | | |
| Yes | 41 (74.5) | 7 (43.8) | 22 (81.5) | 12 (100.0) | 34 (85.0) | 7 (46.7) |
| No | 14 (25.5) | 9 (56.3) | 5 (18.5) | 0 (0.0) | 6 (15.0) | 8 (53.3) |
| **Epilepsy type** | | | | | | |
| *n* | 41 | 7 | 22 | 12 | 34 | 7 |
| Generalized epilepsy | 14 (34.1) | 4 (57.1) | 8 (36.4) | 2 (16.7) | 12 (35.3) | 2 (28.6) |
| Focal epilepsy | 10 (24.4) | 2 (28.6) | 4 (18.2) | 4 (33.3) | 8 (23.5) | 2 (28.6) |
| Combined generalized and focal epilepsy | 16 (39.0) | 0 (0.0) | 10 (45.5) | 6 (50.0) | 13 (38.2) | 3 (42.9) |
| Unknown | 1 (2.4) | 1 (14.3) | 0 (0.0) | 0 (0.0) | 1 (2.9) | 0 (0.0) |
| **Age of onset of first seizure, years** | | | | | | |
| *n* | 41 | 7 | 22 | 12 | 34 | 7 |
| Mean (SD) | 3.0 (4.3) | 1.4 (0.3) | 2.4 (1.6) | 5.0 (7.4) | 1.9 (1.1) | 8.4 (8.5) |
| Median | 1.7 | 1.4 | 1.8 | 2.5 | 1.5 | 4.7 |
| Min–Max | 0.6–25.2 | 1.1–1.7 | 1.0–7.3 | 0.6–25.2 | 0.6–6.2 | 1.0–25.2 |
| **Average seizure frequency before study enrollment** | | | | | | |
| *n* | 41 | 7 | 22 | 12 | 34 | 7 |
| <1 seizure/month | 29 (70.7) | 4 (57.1) | 16 (72.7) | 9 (75.0) | 24 (70.6) | 5 (71.4) |
| <1 seizure/day | 8 (19.5) | 2 (28.6) | 5 (22.7) | 1 (8.3) | 6 (17.6) | 2 (28.6) |
| 1–5 seizures/day | 2 (4.9) | 1 (14.3) | 1 (4.5) | 0 (0.0) | 2 (5.9) | 0 (0.0) |
| 6–10 seizures/day | 2 (4.9) | 0 (0.0) | 0 (0.0) | 2 (16.7) | 2 (5.9) | 0 (0.0) |
| **Seizure type** | | | | | | |
| *n* | 42 | 8 | 22 | 12 | 35 | 7 |
| Focal onset | 26 (61.9) | 2 (25.0) | 14 (63.6) | 10 (83.3) | 21 (60.0) | 5 (71.4) |
| Generalized onset | 30 (71.4) | 4 (50.0) | 18 (81.8) | 8 (66.7) | 25 (71.4) | 5 (71.4) |
| Unknown | 2 (4.8) | 2 (25.0) | 0 (0.0) | 0 (0.0) | 2 (5.7) | 0 (0.0) |
| **Focal onset consciousness** | | | | | | |
| *n* | 26 | 2 | 14 | 10 | 21 | 5 |
| Aware | 9 (34.6) | 1 (50.0) | 5 (35.7) | 3 (30.0) | 7 (33.3) | 2 (40.0) |
| Impaired consciousness | 18 (69.2) | 1 (50.0) | 10 (71.4) | 7 (70.0) | 15 (71.4) | 3 (60.0) |
| **Focal onset type** | | | | | | |
| *n* | 26 | 2 | 14 | 10 | 21 | 5 |
| Motor onset | 22 (84.6) | 1 (50.0) | 12 (85.7) | 9 (90.0) | 18 (85.7) | 4 (80.0) |
| Nonmotor onset | 5 (19.2) | 1 (50.0) | 3 (21.4) | 1 (10.0) | 4 (19.0) | 1 (20.0) |
| **Focal motor onset type** | | | | | | |
| *n* | 22 | 1 | 12 | 9 | 18 | 4 |
| Atonic | 15 (68.2) | 1 (100.0) | 6 (50.0) | 8 (88.9) | 13 (72.2) | 2 (50.0) |
| Clonic | 5 (22.7) | 0 (0.0) | 4 (33.3) | 1 (11.1) | 4 (22.2) | 1 (25.0) |
| Epileptic spasm | 3 (13.6) | 0 (0.0) | 2 (16.7) | 1 (11.1) | 3 (16.7) | 0 (0.0) |
| Myoclonic | 2 (9.1) | 0 (0.0) | 2 (16.7) | 0 (0.0) | 1 (5.6) | 1 (25.0) |
| Tonic | 4 (18.2) | 0 (0.0) | 4 (33.3) | 0 (0.0) | 3 (16.7) | 1 (25.0) |
| **Focal nonmotor onset type** | | | | | | |
| *n* | 5 | 1 | 3 | 1 | 4 | 1 |
| Behavioral arrest | 4 (80.0) | 0 (0.0) | 3 (100.0) | 1 (100.0) | 3 (75.0) | 1 (100.0) |
| Emotional | 1 (20.0) | 1 (100.0) | 0 (0.0) | 0 (0.0) | 1 (25.0) | 0 (0.0) |
| **Not focal onset type** | | | | | | |
| *n* | 26 | 3 | 15 | 8 | 21 | 5 |
| Motor | 16 (61.5) | 2 (66.7) | 2 (66.7) | 4 (50.0) | 13 (61.9) | 3 (60.0) |
| Nonmotor | 3 (11.5) | 0 (0.0) | 2 (13.3) | 1 (12.5) | 2 (9.5) | 1 (20.0) |
| Absence | 10 (38.5) | 3 (100.0%) | 4 (26.7) | 3 (37.5) | 8 (38.1) | 2 (40.0) |
| **Not focal motor onset type** | | | | | | |
| *n* | 16 | 2 | 10 | 4 | 13 | 3 |
| Tonic clonic | 12 (75.0) | 1 (50.0) | 7 (70.0) | 4 (100.0) | 11 (84.6) | 1 (33.3) |
| Other motor tonic | 1 (6.3) | 0 (0.0) | 1 (10.0) | 0 (0.0) | 1 (7.7) | 0 (0.0) |
| Other motor clonic | 1 (6.3) | 0 (0.0) | 1 (10.0) | 0 (0.0) | 1 (7.7) | 0 (0.0) |
| Other motor atonic | 7 (43.8) | 2 (100.0) | 5 (50.0) | 0 (0.0) | 5 (38.5) | 2 (66.7) |
| **Seizure detected on EEG** | | | | | | |
| *n* | 38 | 8 | 20 | 10 | 32 | 6 |
| Seizure detected | 12 (31.6) | 2 (25.0) | 2 (25.0) | 5 (50.0) | 10 (31.3) | 2 (33.3) |
| Seizure not detected | 27 (71.1) | 6 (75.0) | 16 (80.0) | 5 (50.0) | 23 (71.9) | 4 (66.7) |
| For some questions more than one response may be given per subject, resulting in the sum of each category exceeding the small n count. Percentages are based on the small n count.  *AS* Angelman syndrome, *EEG* electroencephalogram, *Max* maximum, *Min* minimum, *SD* standard deviation | | | | | | |

**Table S3** Summary of seizures reported through the app, seizure history at baseline, and participant information

| **Participant genotype** | **Age (years)** | **Days with device** | **Number of seizures reported in diary** | **Frequency reported at baseline** | **Frequency observed in diary** |
| --- | --- | --- | --- | --- | --- |
| Deletion | 26.0 | 463 | 385 | 6–10 seizures/day | <1 seizure/day |
| Deletion | 1.8 | 375 | 53 | <1 seizure/day | <1 seizure/day |
| Deletion | 22.4 | 407 | 26 | 6–10 seizures/day | <1 seizure/day |
| Deletion | 18.2 | 371 | 25 | <1 seizure/month | <1 seizure/day |
| Deletion | 5.5 | 246 | 18 | 1–5 seizures/day | <1 seizure/day |
| Deletion | 12.5 | 360 | 17 | <1 seizure/day | <1 seizure/day |
| Deletion | 8.1 | 246 | 15 | <1 seizure/day | <1 seizure/day |
| Deletion | 4.7 | 319 | 13 | <1 seizure/month | <1 seizure/day |
| Deletion | 29.0 | 379 | 4 | <1 seizure/month | <1 seizure/month |
| Deletion | 7.3 | 435 | 3 | <1 seizure/day | <1 seizure/month |
| Deletion | 2.5 | 361 | 2 | <1 seizure/month | <1 seizure/month |
| Deletion | 1.5 | 461 | 2 | No seizures | <1 seizure/month |
| Deletion | 3.5 | 359 | 1 | <1 seizure/month | <1 seizure/month |
| Deletion | 5.2 | 427 | 1 | <1 seizure/month | <1 seizure/month |
| Deletion | 9.5 | 399 | 1 | <1 seizure/month | <1 seizure/month |
| Deletion | 20.8 | 350 | 1 | <1 seizure/month | <1 seizure/month |
| Deletion | 1.6 | 228 | 0 | <1 seizure/day | No seizures |
| Deletion | 30.7 | 481 | 0 | <1 seizure/day | No seizures |
| Deletion | 2.7 | 409 | 0 | <1 seizure/month | No seizures |
| Deletion | 5.1 | 304 | 0 | <1 seizure/month | No seizures |
| Deletion | 6.7 | 249 | 0 | <1 seizure/month | No seizures |
| Deletion | 7.2 | 242 | 0 | <1 seizure/month | No seizures |
| Deletion | 7.7 | 220 | 0 | <1 seizure/month | No seizures |
| Deletion | 7.8 | 546 | 0 | <1 seizure/month | No seizures |
| Deletion | 8.1 | 330 | 0 | <1 seizure/month | No seizures |
| Deletion | 8.5 | 320 | 0 | <1 seizure/month | No seizures |
| Deletion | 9.9 | 355 | 0 | <1 seizure/month | No seizures |
| Deletion | 10.0 | 410 | 0 | <1 seizure/month | No seizures |
| Deletion | 10.3 | 344 | 0 | <1 seizure/month | No seizures |
| Deletion | 12.8 | 260 | 0 | <1 seizure/month | No seizures |
| Deletion | 18.2 | 384 | 0 | <1 seizure/month | No seizures |
| Deletion | 23.4 | 492 | 0 | <1 seizure/month | No seizures |
| Deletion | 26.3 | 479 | 0 | <1 seizure/month | No seizures |
| Deletion | 27.0 | 393 | 0 | <1 seizure/month | No seizures |
| Deletion | 3.0 | 590 | 0 | 1–5 seizures/day | No seizures |
| Deletion | 1.9 | 484 | 0 | No seizures | No seizures |
| Deletion | 3.0 | 614 | 0 | No seizures | No seizures |
| Deletion | 3.0 | 444 | 0 | No seizures | No seizures |
| Deletion | 3.8 | 280 | 0 | No seizures | No seizures |
| Deletion | 3.8 | 453 | 0 | No seizures | No seizures |
| Nondeletion | 5.6 | 286 | 60 | <1 seizure/day | <1 seizure/day |
| Nondeletion | 18.7 | 498 | 4 | <1 seizure/month | <1 seizure/month |
| Nondeletion | 9.1 | 269 | 0 | <1 seizure/day | No seizures |
| Nondeletion | 9.0 | 294 | 0 | <1 seizure/month | No seizures |
| Nondeletion | 9.1 | 385 | 0 | <1 seizure/month | No seizures |
| Nondeletion | 11.3 | 238 | 0 | <1 seizure/month | No seizures |
| Nondeletion | 37.9 | 400 | 0 | <1 seizure/month | No seizures |
| Nondeletion | 2.7 | 393 | 0 | No seizures | No seizures |
| Nondeletion | 3.4 | 362 | 0 | No seizures | No seizures |
| Nondeletion | 3.4 | 393 | 0 | No seizures | No seizures |
| Nondeletion | 5.5 | 264 | 0 | No seizures | No seizures |
| Nondeletion | 7.0 | 263 | 0 | No seizures | No seizures |
| Nondeletion | 7.5 | 470 | 0 | No seizures | No seizures |
| Nondeletion | 8.7 | 232 | 0 | No seizures | No seizures |
| Nondeletion | 9.9 | 252 | 0 | No seizures | No seizures |
| The frequency reported at baseline is the estimation provided by the caregiver during the initial clinical evaluation (source: eCRF). *eCRF* electronic case report form | | | | | |

**Table S4** Numbers of EEG recordings

|  | **TDC** | **AS deletion** | **AS nondeletion** | **All AS** | **Total** |
| --- | --- | --- | --- | --- | --- |
| **Visit 1** | 13 | 26 | 8 | 34 | 47 |
| **Visit 2** | 8 | 16 | 5 | 21 | 29 |
| **Visit 3** | 4 | 1 | 1 | 2 | 6 |
| **Total** | 25 | 43 | 14 | 57 | 82 |
| *AS* Angelman syndrome, *EEG* electroencephalogram, *TDC* typically developing children | | | | | |

**Table S5** Comparison of Bayley-III data from FREESIAS and the AS-NHS

| Bayley-III domains  Raw scores, *n*, mean ± SD | Study | AS 1–4 years | AS 5–12 years | AS ≥18 years | AS deletion | AS nondeletion |
| --- | --- | --- | --- | --- | --- | --- |
| Cognitive | FREESIAS | *n* = 16  35.5 ± 7.4 | *n* = 26  49.9 ± 11.1 | *n* = 12  50.9 ± 11.6 | *n* = 40  41.5 ± 9.6 | *n* = 14  58.3 ± 10.0 |
|  | AS-NHS | *n* = 149  41.5 ± 10.3 | *n* = 86  51.1 ± 11.4 | *n* = 11  56.5 ± 11.9 | *n* = 168  40.3 ± 8.4 | *n* = 78  56.9 ± 10.4 |
| Expressive Communication | FREESIAS | *n* = 15  9.0 ± 3.7 | *n* = 27  11.9 ± 4.3 | *n* = 12  15.8 ± 5.1 | *n* = 39  10.9 ± 4.3 | *n* = 15  14.7 ± 5.5 |
|  | AS-NHS | *n* = 149  10.4 ± 3.8 | *n* = 85  12.7 ± 5.2 | *n* = 11  12.2 ± 6.4 | *n* = 168  9.5 ± 3.2 | *n* = 77  15.1 ± 4.8 |
| Receptive Communication | FREESIAS | *n* = 16  12.9 ± 4.2 | *n* = 25  17.4 ± 5.6 | *n* = 12  19.3 ± 7.0 | *n* = 39  14.3 ± 4.4 | *n* = 14  22.6 ± 5.8 |
|  | AS-NHS | *n* = 149  13.8 ± 4.5 | *n* = 85  17.6 ± 7.6 | *n* = 11  19.5 ± 8.1 | *n* = 168  12.7 ± 3.5 | *n* = 77  21.4 ± 6.7 |
| Fine Motor | FREESIAS | *n* = 15  24.1 ± 3.8 | *n* = 26  29.6 ± 7.3 | *n* = 12  35.3 ± 8.0 | *n* = 39  27.0 ± 6.5 | *n* = 14  35.9 ± 7.7 |
|  | AS-NHS | *n* = 149  26.6 ± 5.4 | *n* = 88  32.5 ± 8.8 | *n* = 11  36.3 ± 9.2 | *n* = 168  25.8 ± 4.2 | *n* = 79  36.2 ± 8.4 |
| Gross Motor | FREESIAS | *n* = 16  36.6 ± 10.7 | *n* = 27  49.3 ± 6.7 | *n* = 10  48.4 ± 7.6 | *n* = 38  42.8 ± 10.1 | *n* = 15  51.5 ± 6.5 |
|  | AS-NHS | *n* = 149  38.1 ± 9.4 | *n* = 89  48.8 ± 6.6 | *n* = 11  49.6 ± 7.3 | *n* = 168  38.9 ± 8.5 | *n* = 81  49.7 ± 8.5 |
| Comparison of Bayley-III raw scores data from FREESIAS and the Natural History Study for Cognitive, Expressive Communication, Receptive Communication, Fine Motor, and Gross Motor domains.  *AS* Angelman syndrome, *AS-NHS* Angelman Syndrome Natural History Study, *Bayley-III* Bayley Scales of Infant and Toddler Development® – Third Edition, *SD* standard deviation | | | | | | |

**Table S6** Study expectations and output from feedback questionnaires

| Questions and answers, *n* (%) | AS | TDC |
| --- | --- | --- |
| ***In what type of research did you participate before?*** |  |  |
| *n/N* | 54 | 20 |
| Never participated before | 32 (59.3) | 13 (65.0) |
| Research into new medicine or therapy | 5 (9.3) | 1 (5.0) |
| Nondrug study | 13 (24.1) | 4 (20.0) |
| Survey-type study | 12 (22.2) | 4 (20.0) |
| Interview or focus group study | 2 (3.7) | 0 (0.0) |
| Other | 1 (1.9) | 0 (0.0) |
| ***If participated before, how would you describe your experience?*** |  |  |
| *n* | 23 | 7 |
| Positive experience | 15 (65.2) | 6 (85.7) |
| Neutral experience | 7 (30.4) | 1 (14.3) |
| ***For what reasons did you choose to participate in this study?*** |  |  |
| *n/N* | 54 | 20 |
| Help contribute to medical research | 50 (92.6) | 19 (95.0) |
| Learn more about Angelman syndrome | 41 (75.9) | 10 (50.0) |
| Get more treatment options for Angelman syndrome | 44 (81.5) | 12 (60.0) |
| Raise awareness in the community | 22 (40.7) | 8 (40.0) |
| Other | 6 (11.1) | 1 (5.0) |
| **In-clinic assessments, *n* (%):** |  |  |
| ***Did you complete the in-clinic visit over 1 or 2 days?***  *n*  Completed in-clinic visit over 1 day  Completed in-clinic visit over 2 days | 43  26 (60.5)  17 (39.5) | 17  13 (76.5)  4 (23.5) |
| ***How satisfied or dissatisfied are you with your experience of completing the in-clinic assessments?***  *n*  Very satisfied  Satisfied  Neither satisfied nor dissatisfied  Dissatisfied | 43  23 (53.5)  14 (32.6)  5 (11.6)  1 (2.3) | 17  15 (88.2)  2 (11.8)  0 (0.0)  0 (0.0) |
| ***How satisfied or dissatisfied are you with your child’s experience of completing the in-clinic assessments?***  *n*  Very satisfied  Satisfied  Neither satisfied nor dissatisfied | 43  24 (55.8)  11 (25.6)  8 (18.6) | 17  15 (88.2)  2 (11.8)  0 (0.0) |
| ***How would you rate the frequency with which you were asked to complete the clinic visits?***  *n*  Frequency of clinic visits was acceptable | 43  43 (100.0) | 17  17 (100.0) |
| ***How would you rate the length of clinic visits?***  *n*  Length of clinic visits was acceptable  Length of clinic visits was unacceptable (too long) | 43  34 (79.1)  9 (20.9) | 17  17 (100.0)  0 (0.0) |
| ***How would you rate the support provided for you to attend the clinic visits, e.g., travel support and reimbursement?***  *n*  Very satisfied  Satisfied  Neither satisfied nor dissatisfied | 43  30 (69.8)  11 (25.6)  2 (4.7) | 17  15 (88.2)  2 (11.8)  0 (0.0) |
| **At-home visits, *n* (%):** |  |  |
| ***How would you rate the frequency of home visits?***  *n*  Frequency of home visits was acceptable  Frequency of home visits was unacceptable (too frequent) | 38  37 (97.4)  1 (2.6) | 12  12 (100.0)  0 (0.0) |
| ***How would you rate the burden of home visit for your child and family?***  *n*  No burden  Slight burden  Moderate burden  Extreme burden | 38  8 (21.1)  17 (44.7)  11 (28.9)  2 (5.3) | 12  2 (16.7)  5 (41.7)  5 (41.7)  0 (0.0) |
| **Smartphone assessments, *n* (%):** |  |  |
| ***How satisfied or dissatisfied are you with how easy it was to use the smartphone?***  *n*  Very satisfied  Satisfied  Neither satisfied nor dissatisfied  Dissatisfied | 43  13 (30.2)  15 (34.9)  7 (16.3)  8 (18.6) | 17  6 (35.3)  3 (17.6)  2 (11.8)  6 (35.3) |
| ***How would you rate the frequency with which you were asked to complete the smartphone assessments?***  *n*  Frequency of assessment was acceptable  Frequency of assessment was unacceptable (too frequent) | 43  33 (76.7)  10 (23.3) | 17  12 (70.6)  5 (29.4) |
| ***How would you rate the time required to complete the smartphone assessments?***  *n*  Time required to complete assessments was acceptable  Time required to complete assessments was unacceptable (too long) | 43  42 (97.7)  1 (2.3) | 17  16 (94.1)  1 (5.9) |
| ***How satisfied or dissatisfied were you with your experience of completing the at-home EEG assessments?***  *n*  Very satisfied  Satisfied  Neither satisfied nor dissatisfied  Dissatisfied  Very dissatisfied | 37  12 (32.4)  10 (27.0)  9 (24.3)  3 (8.1)  3 (8.1) | 12  5 (41.7)  3 (25.0)  2 (16.7)  2 (16.7)  0 (0.0) |
| ***How much was the sleep of your child impacted by the overnight EEG?***  *n*  Not at all impacted  Slightly impacted  Very impacted  Extremely impacted | 35  5 (14.3)  11 (31.4)  9 (25.7)  10 (28.6) | 12  3 (25.0)  8 (66.7)  1 (8.3)  0 (0.0) |
| ***In future, would you prefer overnight EEG assessments at home or in the clinic?***  *n*  Would prefer assessment in-clinic  Would prefer assessment at home | 40  10 (25.0)  30 (75.0) | 17  6 (42.9)  8 (57.1) |
| **At-home sleep devices, *n* (%):** |  |  |
| ***How satisfied or dissatisfied are you with how easy it was to use the sleep mat?***  *n*  Very satisfied  Satisfied  Neither satisfied nor dissatisfied  Very dissatisfied | 43  29 (67.4)  10 (23.3)  4 (9.3)  0 (0.0) | 17  12 (70.6)  3 (17.6)  1 (5.9)  1 (5.9) |
| ***How satisfied or dissatisfied are you with how easy it was to use the wearable sleep monitor?***  *n*  Very satisfied  Satisfied  Neither satisfied nor dissatisfied  Dissatisfied  Very dissatisfied | 36  13 (36.1)  10 (27.8)  8 (22.2)  4 (11.1)  1 (2.8) | 13  8 (61.5)  3 (23.1)  1 (7.7)  1 (7.7)  0 (0.0) |
| **Study overall, *n* (%):** |  |  |
| ***How satisfied or dissatisfied are you with your experience in the study overall?***  *n*  Very satisfied  Satisfied  Neither satisfied nor dissatisfied  Dissatisfied  Very dissatisfied | 43  16 (37.2)  18 (41.9)  5 (11.6)  1 (2.3)  3 (7.0) | 17  13 (76.5)  4 (23.5)  0 (0.0)  0 (0.0)  0 (0.0) |
| *AS* Angelman syndrome, *EEG* electroencephalogram, *TDC* typically developing children | | |

# Supplementary figures

**Fig. S****1.** Seizures reported via the seizure diary over time


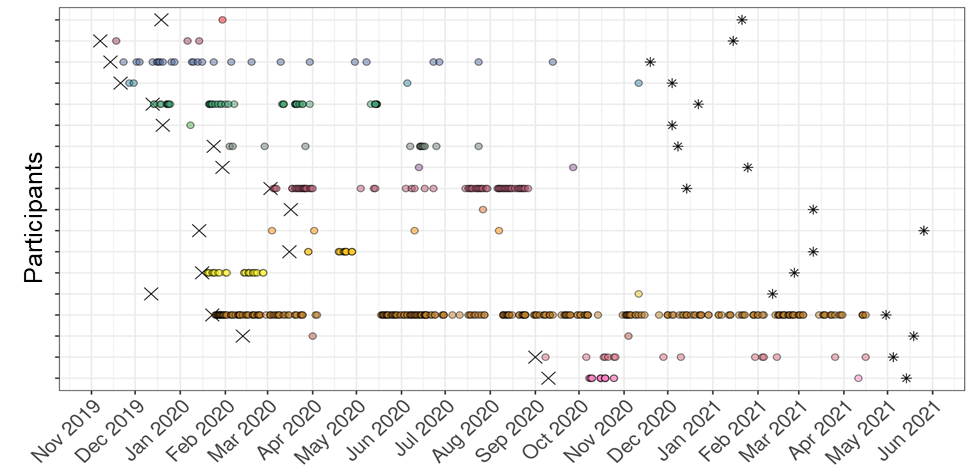


Reported seizure event

Last day on study

Date when the caregiver received the device

Each line shows data from a different participant and each circle represents one reported seizure event. Participants that did not report any seizures through the diary were not included.
